# Patient-related healthcare costs for diarrhoea, Guillain Barré syndrome and invasive non-typhoidal salmonellosis in Gondar, Ethiopia, 2020

**DOI:** 10.1186/s12889-022-14539-1

**Published:** 2022-11-16

**Authors:** Coen P. A. van Wagenberg, T. Guadu Delele, Arie H. Havelaar

**Affiliations:** 1 Wageningen Economic Research, P.O. Box 29703, 2502 LS Den Haag, the Netherlands; 2 Department of Environmental and Occupational Health and Safety, Institute of Public Health, College of Medicine and Health Sciences, University of Gondar, Gondar, Ethiopia; 3 Emerging Pathogens Institute, Animal Sciences Department, Global Food Systems Institute, University of Florida, Gainesville, FL USA

**Keywords:** Healthcare costs, Cost-of-illness, Foodborne disease, Direct, Indirect, Hospital, Health centre, Private clinic, Campylobacter, Salmonella, E. coli, ETEC, Ethiopia

## Abstract

**Background:**

Globally, foodborne diseases result in a significant disease burden with low- and middle-income countries disproportionately affected. Estimates of healthcare costs related to foodborne disease can aid decision makers to take action to mitigate risks and prevent illness. However, only limited data on the African continent are available, especially related to more severe sequelae. We provide estimates of direct and indirect (non)-medical costs of patients with diarrhoea, Guillain-Barré syndrome (GBS), and invasive non-typhoidal salmonellosis (iNTS) in three healthcare facilities in Gondar, Ethiopia.

**Methods:**

We used healthcare data from patient records, interviews with family caregivers and 2020 healthcare resource unit costs. Descriptive statistical analysis was performed. For diarrhoea, differences in mean and median transformed costs between healthcare facilities and etiologies (*Campylobacter* spp., enterotoxigenic *Escherichia coli*, non-typhoidal *Salmonella enterica*) were analysed with ANOVA and chi squared tests. Contribution of healthcare facility, dehydration severity, sex, age and living area to transformed costs was identified with linear regression. Results are in 2020 USD per patient. To extrapolate to national level, 2017 national incidence estimates were used.

**Results:**

Mean direct medical costs were 8.96 USD for diarrhoea (health centre 6.50 USD, specialised hospital 9.53 USD, private clinic 10.56 USD), 267.70 USD for GBS, and 47.79 USD for iNTS. Differences in costs between diarrhoea patients were mainly associated with healthcare facility. Most costs did not differ between etiologies. Total costs of a diarrhoea patient in the specialised hospital were 67 USD, or 8% of gross national income per capita. For direct medical plus transport costs of a GBS and iNTS patient in the specialised hospital, this was 33% and 8%, respectively. Of the 83.9 million USD estimated national non-typhoidal *Salmonella enterica* related cost, 12.2% was due to iNTS, and of 187.8 million USD related to *Campylobacter* spp., 0.2% was due to GBS.

**Conclusion:**

Direct medical costs per patient due to GBS and iNTS were 30 respectively five times those due to diarrhoea. Costs of a patient with diarrhoea, GBS or iNTS can be a substantial part of a household’s income. More severe sequalae can add substantially to cost-of-illness of foodborne hazards causing diarrheal disease.

**Supplementary Information:**

The online version contains supplementary material available at 10.1186/s12889-022-14539-1.

## Background

Globally, foodborne disease (FBD) result in a significant public health impact, with low- and middle-income countries (LMICs), and especially in Africa, affected relatively more than high income countries [1]. In 2010, 600 million cases of foodborne disease were estimated globally resulting in 420,000 deaths [1]. Of the food borne disease related deaths, 230,000 were estimated to be due to foodborne hazards causing diarrheal disease [1]. Particularly in Africa, diarrheal disease agents were estimated to be the leading cause of FBD [1]. The economic burden associated with FBD in LMICs was estimated at 95.2 billion USD per year [2]. This was estimated on the basis of “productivity losses” by multiplying the disease burden associated with disability or premature death captured in disability adjusted life years with gross national income per capita. Of the estimated global burden, LMICs in Asia account for 63.1 billion USD and LMICs in Sub-Saharan Africa for 16.7 billion USD. In addition, costs of treating foodborne illnesses in LMICs were estimated at 15.1 billion USD a year [2]. This was estimated by multiplying the number of new illness cases per year with a rough estimate of costs for medical treatment and out-of-pocket expenses of 27 USD per case. This last estimate was based on only a few studies in different countries. Due to data limitations, both assessments only capture part of the total burden and costs of FBD in LMICs. Improved estimates of the economic burden associated with FBD and treatment costs can inform decision makers in LMICs better about the size of the problem and motivate them more to take action to mitigate risks and prevent illness.

There are several studies available about the economic burden due to foodborne or diarrheal disease on the African continent. For The Gambia, Kenya, and Mali in 2011, direct medical costs of hospitalization due to diarrhoea due to any etiology in children up to 5 years were estimated between 0.70 and 2.20 USD per patient depending on the country, direct non-medical transport costs between 0.19 and 0.55 USD, and indirect non-medical costs due to lost income of caregivers between 1.55 and 4.99 USD [3]. For The Gambia, Mali, Mozambique and Kenya in 2012, mean inpatient total costs related to diarrhoea due to any etiology in children up to 5 years were estimated between 11.03 and 151.95 USD per patient, depending on the country [4]. For Rwanda in 2014, direct hospital costs, transport costs and lost income due to diarrhoea in children up to 5 years were estimated at 44.22, 2.06, and 41.78 USD per patient, respectively [5]. For Burkina Faso in 2011, average out-of-pocket costs among individuals reporting illness or injury in Ouagadougou were estimated at 17.4 USD per episode of illness [6]. Expenditure was higher for those receiving care in private facilities compared to in public ones, and medication was the most expensive component of expenditure. For Burkina Faso in 2013/14 and 2016, out-of-pocket costs related to serious injury and illness were estimated at 137.3 Euro per illness case per household [7]. For Ethiopia in 2016, the median of the sum of direct medical, non-medical and indirect costs in the referral hospital in the city of Gondar plus costs of treatment before the hospital visit were estimated at 22.25 USD per patient coming to the outpatient pharmacy [8]. The above studies focus on diarrhoea or general illness. Although diarrhoea is an important illness related to food safety hazards, it causes only part of costs associated with food safety hazards. Food safety hazards can also result in sequalae and associated illness costs, as shown for example for the Netherlands [9]. For the African context, information about illness costs related to such sequalae are lacking.

This study aims to help fill this gap by gathering data about the illness costs related to sequalae associated with foodborne diarrheal disease agents selected by the project “Urban Food Markets in Africa – incentivizing food safety (Pull-Push Project)” in Ethiopia (https://www.ilri.org/research/projects/urban-food-markets-africa-incentivizing-food-safety-using-pull-push-approach): *Campylobacter* spp., enterotoxigenic *Escherichia coli* (ETEC) and non-typhoidal *Salmonella enterica*. To estimate the disease burden related to foodborne pathogens, the World Health Organization (WHO) Foodborne Disease Burden Epidemiology Reference Group (FERG) used a hazard-based approach based on health states [10]. They defined health states as symptoms and sequelae including death that are causally related to the concerned hazard and transmitted through food. In our study, following the FERG approach, a health state of interest for all three disease agents is diarrhoea. For *Campylobacter* spp., we also included Guillain-Barré syndrome (GBS), an autoimmune disease that can be triggered by different infectious agents, including *Campylobacter jejuni*, when specific surface antigens mimic gangliosides in the host nervous system [11]. The FERG estimates were based on a review suggesting 31% of GBS cases globally were attributable to *Campylobacter* infection [12]. Infection caused by non-typhoidal *Salmonella enterica* may also lead to invasive non-typhoidal salmonellosis (iNTS) with the highest incidence reported in Africa [13]. Data about healthcare resource use and associated costs related to these health states in sub-Saharan Africa, and specifically in Ethiopia, are scarce or lacking. This study aimed to provide estimates of healthcare costs due to diarrhoea, GBS and iNTS in Ethiopia.

Healthcare costs can be divided into direct and indirect costs and medical and non-medical costs. Direct costs are related to treatment of the disease itself, such as costs of healthcare resource used and of travel, accommodation and meals during the illness period. Indirect costs are costs related to lost earnings and productivity, but also to medical costs incurred in the life years gained because of treating a disease, but that are unrelated to that disease. Medical costs are costs made within the healthcare system, non-medical costs those made outside the healthcare system. In our study, we adopted the societal perspective focusing on size of costs rather than who pays them. Following standard practice [9, 14], we included direct medical costs, direct non-medical costs and indirect non-medical costs. Note that indirect medical costs were not included in our study.

## Methods

### Health care facilities

Healthcare in Ethiopia is a mix of public, for-profit private, and not-for-profit private initiatives and institutes. The public healthcare system in Ethiopia has three tiers, categorized according to their population coverage [15]. At tier one, health posts serve a population of 5,000, health centres of 25,000, and primary hospitals of 100,000. At tier two, general hospitals serve a population of one million. At tier three, specialised hospitals serve a population of five million. In addition, traditional healers, herbalists, and pharmacies as well as private for-profit and not-for-profit healthcare facilities such as specialty clinics can deliver healthcare. Private healthcare clinics and hospitals can specialize on specific healthcare services, depending on market demand, and come in different sizes in terms of healthcare capacity and population coverage [15]. Due to budget limitations, we collected data in one facility from each tier in the city of Gondar: the Maraki health centre as an example from tier one, a private specialty clinic with a population coverage like a general hospital in tier two, and the University of Gondar referral hospital as an example of a specialised hospital from tier three. Consequently, the data collected in our study can only provide an indication of the healthcare resource use and associated costs for the whole country.

### Health care resource use and unit costs

Table 1 provides the data collection strategy per healthcare facility. Data for direct medical and direct non-medical costs due to diarrhoea, GBS and iNTS were collected from paper patient records available in the healthcare facilities’ registration systems. For diarrhoea, GBS and iNTS, patients with final diagnosis diarrhoea, GBS and invasive non-typhoidal salmonellosis were selected, respectively. For diarrhoea, we aimed to collect 100 records of patients in the outpatient ward in each healthcare facility and 100 records of patients in the inpatient wards in the specialised hospital, in total 400 records. In the outpatient ward of the specialised hospital, this was approximately one third of the annual number of patients diagnosed with diarrhoea, in the Maraki health centre approximately half, and in the private clinic and inpatient wards of the specialized hospital approximately all. We collected records of patients admitted in the year preceding the data collection period. After chronologically ordering on discharge date, we selected every third record of patients diagnosed with diarrhoea in the outpatient ward of the specialised hospital and every second record in the Maraki health centre. All records of patients diagnosed with diarrhoea in the outpatient ward of the private clinic and inpatient wards of the specialized hospital were collected. Incomplete records were removed from our dataset and excluded from the analyses. To account for this, we collected 5% more records than aimed for (420 in total). Data concerned patients discharged between 20 and 2019 and 15 August 2020. For GBS and iNTS, we collected the records of all patients admitted to the specialised hospital in the three years preceding the data collection period, due to the lower incidence. For GBS, data concerned patients discharged between 11 and 2016 and 2 June 2020, and for iNTS between 18 and 2016 and 24 May 2020.

Data for direct medical and indirect non-medical costs due to diarrhoea were collected in face-to-face interviews with family caregivers of people with diarrhoea entering the specialised hospital. Data was extracted anonymously using data collection protocols (Supplementary material S1). Our protocols were designed based on the standardized data collection protocols to estimate the economic burden of diarrhoea disease due to rotavirus developed by the WHO [16]. Questions concerning facility and patient information were mostly copied from the WHO protocols. Questions concerning the treatment history were adapted to the diseases of interest (diarrhoea, GBS, iNTS), for example those concerning final diagnosis, special services, diagnostic tests, other treatments and specialist consultations. A draft version of the protocols was pre-tested with physicians of the University of Gondar referral hospital to check for correctness, completeness and cultural appropriateness. Data were collected in 2020 by a one of the co-authors (TG). Data collection started after ethical clearance was received from the Institutional Ethical Review Board of University of Gondar (V/P/RCS/05/1735/2020) and from the Social Sciences Ethics Committee of Wageningen University and Research (09215846).

**Table 1:** Data collection strategy per healthcare facility for diarrhoea, Guillain-Barré syndrome (GBS) and invasive non-typhoidal salmonellosis (iNTS)

Facility	Tier system		Owner	Diarrhoea		GBS	iNTS
	**Tier**	**Facility type**		**Out- and inpatient**	Family caregiver	**Inpatient**	**Inpatient**
Maraki health centre	1	Health centre	Public	Patient records	N.c. ^b^	N.c	N.c
Speciality clinic^a^	2	Private clinic	Private	Patient records	N.c	N.c	N.c
University of Gondar referral hospital	3	Specialised hospital	Public	Patient records	Family caregiver interviews	Patient records	Patient records

^a^No official tier-system exists for private clinics. The population coverage of the specialty clinic was comparable to that of a general hospital in tier two

^b^Not collected

Data for direct medical costs from patient records included data about the patient (such as age, sex, living area, date of admission and discharge, discharge outcome, admission diagnosis, comorbidities), about the illness history before coming to the healthcare facility (duration of illness, other facilities visited), and about healthcare resources used at the facility related to direct medical costs that the patient was responsible for (co-)paying (diagnostic tests, drugs, length of stay, specialised services). Data for direct non-medical costs included ambulance service, because an ambulance is often used as a transport mode to bring a patient to the healthcare facility rather than a vehicle to provide emergency care. An ambulance is preferred, because most people do not own a car, public transport is often unsuitable to bring a patient to the hospital, and a taxi is often more expensive.

Data for direct medical costs from family caregiver interviews included direct healthcare costs made prior to and during the visit to the hospital and data for indirect non-medical costs lost income. After a physician notified the data collector when a patient with diarrhoea was being treated, the data collector contacted the family caregiver while waiting during the treatment. Interviews were held in a private setting. Informed consent was obtained orally from all interviewed caregivers. The data collector noted the patient record number to later collect the date of discharge. Interviews were held in July 2020.

The data collector gathered facility specific costs per unit of the healthcare resources in Ethiopian Birr (ETB) as used in the healthcare facilities in August 2020 (supplementary material S2). Calculations were performed in ETB and results were converted to United States Dollar (USD) using the World Bank’s 2020 average exchange rate of 34.93 ETB per USD (The World Bank, indicator code PA.NUS.FCRF). Results are presented in 2020 USD.

### Statistical analysis

Frequency distributions were used to present healthcare resource use. Healthcare costs per patient were calculated by multiplying the healthcare resource use per patient with the unit costs per healthcare resource. Not using a healthcare resource use was treated as costs of 0 USD. Within the total direct medical costs, we distinguished the costs categories stay costs, drug costs, diagnostic test costs and special services costs. Special service costs were further divided into special diet costs, specialist consultation costs and intravenous fluids costs. Direct non-medical costs included costs of transportation. Indirect non-medical costs included lost income. Mean, standard deviation, median, minimum, maximum, and 2.5% and 97.5% percentile of costs are presented.

Analysis of differences in mean and median of costs between type of healthcare facility and between etiology of interest in our study (*Campylobacter* spp., ETEC, non-typhoidal *Salmonella enterica*), i.e., the majority of patients in our dataset, was performed for diarrhoea. For GBS and iNTS, data were only available from the specialised hospital. QQ-plot analysis showed costs were not normally distributed. Log_10_ transformation was used for total costs, stay costs, and drug costs. For diagnostic test costs and special services costs x^0.5^ and x^0.75^ were used, respectively, because several patients had no costs in these categories. ANOVA was used to compare mean transformed costs. Levene’s test was used to check for the homogeneity of the variance between groups. If variances were not equal, Tamhane’s T2 test was used as post-hoc test to compare the means between groups, otherwise a Fisher’s Least Significant Difference test was used. Medians of the transformed costs were compared using chi squared tests. A pairwise comparison with an adjusted *p*-value using the false discovery rate method was used as post-hoc test.

Unit costs per healthcare resource differed between healthcare facilities as well as proportion of patients in each category distinguished within sex (female, male), age group (under 5 years, 5 years or older), living area (rural, urban), and severity of dehydration (no, some, severe dehydration). Therefore, linear regression was used to identify the contribution of each in addition to healthcare facility type to each category of transformed costs:

$$Transformed\;costs=constant+\beta_1\cdot{dummy}_{health\;center}+\beta_2\cdot{dummy}_{private\;hospital}+\beta_3\cdot{dummy}_{no\;dehydration}+\beta_4\cdot{dummy}_{some\;dehydration}+\beta_5\cdot female+\beta_6\cdot\;5\;years\;or\;older+\beta_7\cdot rural\;living\;area+\epsilon$$

The specialised hospital, severe dehydration, male, under 5 years, and urban living area were used as baseline in the regression analyses.

All analyses were performed in IBM SPSS Statistics 25, except for the post-hoc median tests which were performed in R-package “rcompanion” [17].

### National cost-of-illness estimates

National cost-of-illness due to FBD caused by *Campylobacter* spp., ETEC and non-typhoidal *Salmonella enterica* in Ethiopia were estimated by multiplying estimated mean direct medical and (in)direct non-medical cost estimates per patient with diarrhoea, GBS or iNTS with 2017 national incidence estimates. Diarrhoea incidences were retrieved from Havelaar, et al. [18]. GBS and iNTS incidence were estimated using the age-weighted probabilities of developing GBS and iNTS as sequelae to diarrhoea by *Campylobacter* spp. and non-typhoidal *Salmonella enterica*, respectively, which were extracted from the FERG database.

## Results

### Diarrhoea

Of the 420 records of patients diagnosed with diarrhoea we collected, records of 406 patients were complete. Of these, roughly half were from the specialised hospital (205), and a quarter each from the private clinic (93) and the health centre (108) (Table 2). At admission, almost 90% of patients were under five years of age. Mean age at admission was 6.2 years in the health centre, and approximately two years in the private and specialised hospital. Approximately 60% of patients were male and almost 80% came from an urban area. In the private clinic, relatively more patients were male (80%) and in the specialised hospital relatively fewer patients were male (44%). The distribution of living area was similar for the healthcare facilities.

**Table 2:** Sex, living area (number of patients) and age at admission (years) of diarrhoea patients in three healthcare facility types in Gondar, Ethiopia, in 2020

Characteristic	Health centre		Private clinic		Specialised hospital		Total	
	< 5 years	≥ 5 years	< 5 years	≥ 5 years	< 5 years	≥ 5 years	< 5 years	≥ 5 years
*Sex*								
Female	42	15	19	0	84	6	145	21
Male	44	7	64	10	102	13	210	30
*Living area*								
Urban	72	14	64	8	151	11	287	33
Rural	14	8	19	2	35	8	68	18
*Age at admission*								
Mean	6.2		2.1		2.0		3.2	
Standard deviation	11.2		2.5		2.1		6.3	
Range	0–68		0–12		0–13		0–68	

Here, we provide a summary of the healthcare resource use for diarrhoea, details can be found in supplementary material S3. Before entering the healthcare facility, patients had on average 2.3 days of diarrhoea, with a median of 2.0 days and a range between zero and fourteen days. Over 80% had between zero and three days of diarrhoea before visiting the healthcare facility. Patients visiting the private clinic had less days of diarrhoea before visiting the healthcare facility (mean 0.9, median 1.0 day) than the specialised hospital (mean 2.7, median 2.0 days) or the health centre (mean 2.8, median 3.0 days).

Duration of stay varied between one and ten days, with 75% staying for one day. Mean stay was lowest in the health centre (1.2 days) and highest in the specialised hospital (1.5 days). Only in the specialised hospital, seven patients stayed more than three days. It should be noted that in our study admittance into the healthcare facility from two hours up to 24 h was recorded as stay of one day, because the fee was the same for all these patients. In all three healthcare facilities, most patients were treated in the paediatric ward or outpatient clinic.

Supportive drugs and antibiotics were used in many patients in all three healthcare facilities, but the type differed. In the health centre, the only supportive drug used was oral rehydration solution (ORS), whereas in the private clinic glucose 40% and metoclopramide and in the specialised hospital Ringer’s solution were used in addition to ORS. Antibiotics were used for most patients in the health centre and private clinic, and for approximately half of the patients in the specialised hospital. In the health centre, the most used antibiotics were cotrimoxazole and metronidazole, in the private clinic ceftriaxone and ampicillin, and in the specialised hospital ceftriaxone and cotrimoxazole. Paracetamol was used more in the health centre than in the other two facilities. Both steroidal (dexamethasone) and non-steroidal (diclofenac) anti-inflammatory drugs were used in the private clinic, not in the other two facilities. Supportive drugs for the gastro-intestinal tract were used in the health centre and the private clinic. A few patients in the health centre and specialised hospital received anthelmintics.

At least one stool test was used for more than 90% of the patients in all three healthcare facilities. At least one blood test was used for 70% of the patients in the private and specialised hospital, whereas in the health centre it was hardly used. A urinalysis was used for approximately 50% of the patients in the specialised hospital, but for less than 5% in the private clinic and the health centre. Blood culture was only used for 5% or less of the patients in all three facilities.

Specialist consultation was used for 11% of patients in the private clinic, 21% of patients in the health centre, and 27% of patients in the specialised hospital. Intravenous fluids were provided to approximately 75% of patients in the health centre and private clinic, and to 88% in the specialised hospital. Special diet was only provided to 24% of patients in the health centre and to 16% of patients in the specialised hospital, but it was not provided in the private clinic.

Mean total direct medical costs over the three healthcare facilities were 8.96 USD per diarrhoea patient (Table 3). Mean total direct medical costs in the health centre of 6.50 USD per patient were significantly lower (*p* < 0.001) than in the specialised hospital of 9.53 USD per patient or in the private clinic of 10.56 USD per patient. Median total direct medical costs were lowest in the health centre with 6.56 USD per patient (p < 0.001), followed by the specialised hospital with 9.16 USD per patient, and the private clinic with 10.02 USD per patient. This was caused by significant differences in special services costs, diagnostic test costs and stay costs between the healthcare facilities. Mean drug costs did not differ significantly between the healthcare facilities, but the median drug costs were significantly lower in the private clinic compared to the other two facilities (*p* = 0.001). In contrast, mean and median stay costs were higher in the private clinic compared to in the other two facilities (*p* < 0.001). Across all types of healthcare facilities, drug costs were highest, followed by special services costs, diagnostic testing costs and stay costs. The higher mean special services costs in the private clinic were caused by higher costs for intravenous fluids. Intravenous fluids made up approximately 80–90% of the total special services costs in all three healthcare facilities. Patients in the private clinic did not use a special diet. Distributions of the patient costs for special diet, specialist consultation, and intravenous fluids all differed significantly (*p* < 0.001) between the healthcare facility types.

**Table 3:** Direct medical costs in 2020 USD per diarrhoea patient in three healthcare facility types in Gondar, Ethiopia, in 2020

	**Costs/patient (2020 USD)**							**N with costs > 0**
**Cost category**	**Mean**^**a**^	**St. dev**	**Median**^**b**^	**Minimum**	**Maximum**	**2.5%**	**97.5%**	
*Health centre* (*N* = 108)								
Stay costs^c^	0.53 a	0.21	0.43 a	0.43	1.29	0.43	1.00	108
Drug costs	3.56 a	1.60	3.44 a	0.92	9.96	1.07	6.08	108
Diagnostic test costs	0.81 a	0.34	0.72 a	0.00	2.58	0.00	1.43	104
Special services costs	1.59 a	0.91	1.57 a	0.00	3.72	0.00	3.52	97
Total costs	6.49 a	1.88	6.54 a	2.29	13.54	3.19	9.71	108
*Private clinic* (*N* = 93)								
Stay costs^c^	1.86 b	0.78	1.43 b	1.43	4.29	1.43	4.29	93
Drug costs	3.55 a	4.62	2.23 b	0.92	31.23	1.28	12.60	93
Diagnostic test costs	2.17 b	0.69	2.43 b	0.00	3.72	1.119	3.72	92
Special services costs	2.99 b	2.14	4.01 b	0.00	8.02	0.00	6.21	70
Total costs	10.56 b	5.05	10.02 b	2.66	39.11	4.27	19.49	93
*Specialised hospital* (*N* = 205)								
Stay costs^c^	0.84 c	0.68	0.57 a	0.57	5.73	0.57	2.29	205
Drug costs	3.65 a	2.12	3.15 a	0.29	14.31	0.57	8.58	205
Diagnostic test costs	2.81 c	1.53	2.86 c	0.00	10.59	0.86	6.56	200
Special services costs	2.22 b	1.07	2.29 c	0.00	6.87	0.00	4.29	198
Total costs	9.53 b	3.45	9.16 c	2.86	26.80	4.32	18.60	205
*All healthcare facilities* (*N* = 406)								
Stay costs^c^	0.99	0.79	0.57	0.43	5.73	0.43	2.86	406
Drug costs	3.60	2.79	3.15	0.29	31.23	0.57	9.10	406
Diagnostic test costs	2.13	1.42	2.00	0.00	10.59	0.72	5.69	396
Special services costs	2.23	1.44	2.00	0.00	8.02	0.00	6.01	365
Total costs	8.96	3.89	8.35	2.29	39.11	3.72	16.95	406

^a^Different letters between rows with the same cost category (for example, rows with stay costs) indicate a difference in means at a level of *p* < 0.05 between the healthcare facility as tested with ANOVA and Tamhane’s T2 test or Fisher's Least Significant Difference test as post-hoc test

^b^Different letters between rows with the same cost category (for example, rows with stay costs) indicate a difference in medians at a level of *p* < 0.05 between the healthcare facilities as tested with Chi square test and a pairwise comparison with adjusted *p*-value as post-hoc test

^c^Only out-of-pocket costs patients had to pay themselves per day they stayed in the healthcare facility. These do not cover total costs for staying one day in the healthcare facility

Differences in total costs and in stay costs between patients with diarrhoea were associated with the healthcare facility (Table 4). Patients in the health centre had the lowest total and stay costs and in the private clinic the highest. Sex, age group, living area and severity of diarrhoea did not contribute to differences in these costs. Differences in drug costs were associated with age, with patients of five years or older having lower drug costs, although this only explained less than two per cent of variation. Patients treated in the health centre had the lowest diagnostic test costs and in the specialised hospital the highest, and patients coming from a rural living area had higher diagnostic test costs than those in the other facilities. Finally, differences in special service costs were associated with the healthcare facility and severity of dehydration, with patients treated in the health centre having the lowest and specialised hospital the highest costs and costs increasing with the severity of dehydration.

**Table 4:** Linear regression results of healthcare facility type, severity of dehydration, sex, age group and living area on each type of the transformed direct medical costs of patients with diarrhoea from three healthcare facility types in Gondar, Ethiopia, in 2020

	**Log**_**10**_**(Total costs)**		**Log**_**10**_**(Stay costs)**^**a**^		**Log**_**10**_**(Drug costs)**		**(Diagnostic test costs)**^**0.5**^		**(Special services costs)**^**0.75**^	
**Variable **^**b**^	**β**^**c**^	**p**	**β**^**c**^	**P**	**β**^**c**^	**P**	**β**^**c**^	**p**	**β**^**c**^	**p**
Constant		0.000		0.000		0.000		0.000		0.000
Health centre	-0.408	0.000	-0.286	0.000	0.042	0.435	-0.689	0.000	-0.157	0.002
Private clinic	0.081	0.093	0.614	0.000	-0.063	0.241	-0.135	0.001	0.115	0.025
No dehydration	-0.192	0.072	0.087	0.272	-0.083	0.484	-0.027	0.771	-0.427	0.000
Some dehydration	-0.189	0.075	0.067	0.396	-0.146	0.214	-0.147	0.111	-0.242	0.030
Female	0.030	0.513	-0.002	0.952	0.043	0.401	0.000	0.991	-0.003	0.956
5 years or older	-0.053	0.249	0.018	0.594	-0.126	0.013	0.052	0.189	-0.020	0.681
Rural living area	0.062	0.167	0.048	0.155	0.046	0.359	0.091	0.019	-0.011	0.825
Adjusted R Square	0.200		0.559		0.014		0.402		0.115	
F-value	15.470		74.324		1.818		39.859		8.488	
p	0.000		0.000		0.082		0.000		0.000	

^a^Only out-of-pocket costs patients had to pay themselves for each day they stayed in the healthcare facility. These do not cover total costs for a hospital day

^b^Specialised hospital, severe dehydration, male, under 5 years and urban living area were baseline dummies

^c^Standardized coefficient

Mean total direct medical costs over the three healthcare facilities did not differ between *Campylobacter* spp., ETEC and non-typhoidal *Salmonella enterica* (Table 5). Mean special service costs for ETEC were higher than for non-typhoidal *Salmonella enterica* and mean diagnostic testing costs for *Campylobacter* spp. were higher than for the other two pathogens. In the health centre and private clinic, mean total costs related to diarrhoea did not differ between the pathogens, except for mean special service costs in the private clinic that were higher for ETEC than for non-typhoidal *Salmonella enterica*. In the specialised hospital, mean total costs for *Campylobacter* spp. were higher than those for non-typhoidal *Salmonella enterica* but did not differ from those for ETEC. This was caused by the higher diagnostic testing and stay costs. Median costs did not differ between etiology, except for the median diagnostic testing costs in the specialised hospital for non-typhoidal *Salmonella enterica* that were lower than those for the other two pathogens (supplementary material S4).

**Table 5:** Mean direct medical costs in 2020 USD per diarrhoea patient by etiology in three healthcare facility types in Gondar, Ethiopia, in 2020

		**Etiology**^**a,b**^			
**Healthcare facility**	**Cost category**	***Campylobacter* spp.**	**Enterotoxigenic *Escherichia coli***	**Non-typhoidal *Salmonella enterica***	***p*-value**^**d**^
All facilities	Number of patients	56	91	142	
	Total costs	10.62	9.23	8.92	0.058
	Stay costs	1.34	0.99	1.11	0.091
	Special services costs	2.57 ab	2.66 a	2.18 b	0.026
	Diagnostic test costs	2.73 a	2.20 b	2.11 b	0.031
	Drug costs	3.98	3.39	3.52	0.609
Health centre	Number of patients	10	22	25	
	Total costs	6.16	6.30	6.73	0.846
	Stay costs	0.52	0.46	0.63	0.826
	Special services costs	1.60	1.80	1.72	0.903
	Diagnostic test costs	0.72	0.89	0.77	0.297
	Drug costs	3.32	3.15	3.75	0.513
Private clinic	Number of patients	17	24	50	
	Total costs	11.68	10.88	9.85	0.119
	Stay costs	2.03	1.66	1.43	0.252
	Special services costs	2.98 ab	3.95 a	2.52 b	0.021
	Diagnostic test costs	2.23	2.29	2.06	0.264
	Drug costs	4.44	2.98	3.38	0.800
Specialised hospital	Number of patients	29	45	67	
	Total costs	11.54 a	9.79 ab	8.99 b	0.024
	Stay costs^c^	1.23	0.89	0.72	0.021
	Special services costs	2.66	2.38	2.09	0.062
	Diagnostic test costs	3.72 a	2.81 b	2.63 b	0.004
	Drug costs	3.92	3.72	3.55	0.638

^a^Different letters between columns indicate difference in means of transformed costs between pathogens at *p* < 0.05, tested with Tamhane’s T2 test or Fisher's Least Significant Difference test

^b^*Campylobacter* spp., ETEC and non-typhoidal *Salmonella enterica* were the indicated cause for the majority of diarrhoea patients (supplementary material S3). Other etiologies were not analysed separately

^c^ANOVA showed a significant difference, but post-hoc Tamhane’s T2 test did not show significant differences in means between pathogens

^d^ANOVA was used to test for differences between pathogens in transformed costs

All patients visited a health provider before they visited the specialised hospital (Table 6). A pharmacy was visited most, followed by a health centre and a shop (no further specification provided in the data collection tool). All respondents indicated to have made costs for drugs, the vast majority for diagnostic tests and approximately half for consultation. Most also made other financial costs, i.e., all other costs than those for drugs, diagnostic tests or consultation. On average, respondents indicated to have had costs of 13.60 USD per patient prior to visiting the specialised hospital, of which 65% were related to drugs, 20% to diagnostic tests, 15% to other financial costs and 10% to consultations. The highest costs were made in a private hospital, followed by pharmacy, health centre, private clinic, traditional healer and shop.

**Table 6:** Direct medical costs made at healthcare providers visited before coming to a specialised hospital in 2020 USD per diarrhoea patient in a specialised hospital in Gondar, Ethiopia, in 2020

**Healthcare provider visited before**		**Drug costs**	**Diagnostic test costs**	**Consultation costs**	**Other financial costs**	**Total costs**
Private hospital	N	4	4	3	4	4
	mean (st.dev)	0.86 (1.98)	0.46 (1.12)	0.2 (0.52)	0.23 (0.49)	1.78 (3.98)
	min–max	0—7.16	0—4.29	0—1.43	0—1.43	0.00 – 14.32
Health centre	N	8	8	3	8	8
	mean (st.dev)	1.06 (1.55)	0.6 (0.83)	0.14 (0.37)	0.31 (0.46)	2.12 (2.98)
	min–max	0—4.58	0—2.29	0—1.15	0—1.15	0.00 – 7.73
Private clinic	N	6	6	4	4	6
	mean (st.dev)	2.46 (4.67)	1.03 (1.77)	0.23 (0.49)	0.26 (0.54)	3.95 (6.87)
	min–max	0—17.18	0—5.73	0—1.43	0—1.43	0.00 – 22.90
Pharmacy	N	17	5	0	11	17
	mean (st.dev)	2.86 (2.49)	0.29 (0.57)	0 (0)	0.57 (0.6)	3.72 (2.98)
	min–max	0—8.59	0—1.72	0—0	0—1.72	0.00 – 10.02
Traditional healer	N	5	1	1	2	5
	Mean (st.dev)	0.94 (2.23)	0.06 (0.31)	0.06 (0.31)	0.11 (0.4)	1.20 (2.69)
	min–max	0—8.59	0—1.43	0—1.43	0—1.43	0.00 – 8.59
Friend	N	0	0	0	0	0.00
	Mean (st.dev)	0 (0)	0 (0)	0 (0)	0 (0)	0.00 (0.00)
	min–max	0—0	0—0	0—0	0—0	0.00 – 0.00
Shop	N	7	3	0	2	7
	Mean (st.dev)	0.6 (0.94)	0.14 (0.37)	0 (0)	0.06 (0.23)	0.83 (1.29)
	min–max	0—2.58	0—1.43	0—0	0—0.86	0 – 3.72
Other	N	0	0	0	0	0.00
	Av. (st.dev)	0 (0)	0 (0)	0 (0)	0 (0)	0.00 (0.00)
	min–max	0—0	0—0	0—0	0—0	0.00 – 0.00
All providers	N	21	19	11	18	21
	Mean (st.dev)	8.82 (5.55)	2.58 (1.52)	0.66 (0.66)	1.55 (1.06)	13.60 (7.07)
	min–max	3.44—26.62	0—5.73	0—1.43	0—4.29	5.44 – 35.22

Mean ambulance costs were 7.22 USD per patient (Table 7). Ambulance costs per patient were significantly (*p* < 0.001) higher in the health centre (16.29 USD) than in the specialised hospital (5.70 USD). The private clinic did not have an ambulance service. Two third of the patients in the health centre and a quarter of the patients in the specialised hospital used ambulance services.

**Table 7:** Direct non-medical costs (ambulance service) in 2020 USD per diarrhoea patient in three healthcare facility types in Gondar, Ethiopia, in 2020

	**Costs/patient (2020 USD)**							**N with costs > 0**
**Facility type**	**Mean**^**a**^	**St. dev**	**Median**^**a**^	**Mini-mum**	**Maxi-mum**	**2.5%**	**97.5%**	
All (*N* = 406)	7.22	12.40	0.00	0.00	68.71	0.00	42.95	119
Health centre (*N* = 108)	16.29 a	14.95	21.47 a	0.00	42.95	0.00	42.95	66
Private clinic (*N* = 93)	0.00 b	0.00	0.00 b	0.00	0.00	0.00	0.00	0
Specialised hospital (*N* = 205)	5.70 c	10.85	0.00 b	0.00	68.71	0.00	34.36	53

^a^Different letters between rows within each comparison indicate a difference between healthcare facility type at a level of *p* < 0.05

Family caregivers indicated mean costs of bringing a diarrhoea patient to the specialised hospital of 3.60 USD for a roundtrip. Half of them used a taxi to bring the patient to the hospital. Taxi and foot trips were used for shorter travelling times, bus/train and car trips for travelling times exceeding one hour. The latter were more expensive than taxi or foot trips. Similar means of transport were used to visit the patient as to bring the patient to the hospital. Respondents indicated that between one and four people made a roundtrip to visit the patient, but this could include multiple people in one visit and one person in multiple visits. Between two and five person roundtrips were made for bringing and visiting a patient. The Pearson correlation coefficient between number of person roundtrips for visiting a patient and duration of stay in the hospital was 0.15. Mean transport costs for bringing and visiting a patient were 13.54 USD per patient with diarrhoea (Table 8). All 21 respondents indicated to have lost income, on average 29.12 USD per patient (Table 8)

**Table 8:** Direct (transport costs) and indirect non-medical costs (lost income) in 2020 USD per diarrhoea patient in a specialised hospital in Gondar, Ethiopia, in 2020

Cost category	Mean	St.dev	Median	Minimum	Maximum	2.5%	97.5%
Transport costs	13.54	26.09	6.87	0	123.68	0.57	73.29
Lost income	29.12	28.19	17.18	4.01	100.20	5.44	93.04

### Guillain-Barré syndrome

Records of 21 patients with GBS were collected in the specialised hospital. Most patients were male and approximately half came from a rural area (Table 9). At admission, five patients were between 20 and 30 years of age, twelve between 30 and 60 years and four between 60 and 70 years (mean 42 years).

**Table 9:** Sex, area of residence and age of patients with GBS in a specialised hospital in Gondar, Ethiopia, in 2020

Characteristic	Unit	Answer	Value
Sex	N	Male	19
		Female	2
Area of residence	N	Urban	7
		Rural	11
		Unknown	3
Age at admission	Years	Mean	41.7
		Standard deviation	15.3
		Range	20–67

Here, we provide a summary of the healthcare resource use of GBS patients, details are provided in supplementary material S3. On discharge, nine patients were alive and well, six partially recovered, three discharged with medical advice, one was referred and two had died. On average, patients were ill for twelve days before entering the hospital (range 0 to 30 days). Cause, signs and symptoms of the illness were not mentioned on the patient records. Seventeen patients received care before entering the hospital, all from only one provider. Most used providers were health centre, primary hospital and private health institution. Ten patients had comorbidities, of which three had two or three. The most common comorbidities were related to the respiratory tract, such as pneumonia and acute respiratory distress syndrome. Patients stayed on average 22.1 days in the hospital, of which 18.2 days in the intensive care unit and 3.9 days in the emergency room. All patients used specialist consultation (one to eight times). Most patients used a special diet and intravenous fluids, mostly only once. Cardiopulmonary resuscitation was used on one patient. All patients used at least one diagnostic test, with full blood count, blood electrolytes, blood glucose and X-ray used most. Most diagnostic tests were applied once per patient. Both intravenous immunoglobulin (IVIg) and methyl prednisolone treatment were given to two patients. All patients consulted a neurologist (one to ten times), 20 patients a physical therapist (two to 28 times) and 19 a nurse (three to 30 times). All patients received antibiotics and most anti-inflammatory drugs as well. Different antibiotics (azithromycin, cefepime, ceftazidime, ceftriaxone, metronidazole, vancomycin) were administered, with half of the patients receiving ceftriaxone. Different steroidal (dexamethasone, prednisolone) and non-steroidal (acyclovir, diclofenac) anti-inflammatory drugs were administered, with half of the patients receiving dexamethasone. Approximately half of the patients received supportive drugs, mainly unfractionated heparin, and supportive drugs for the gastrointestinal tract. Some patients received antidepressant and pain relief drugs. Three patients experienced complications during their hospital stay, one cardiac arrest, one acute respiratory distress syndrome and one deep venous thrombosis. Most patients used ambulance services, mostly only once.

Mean total direct healthcare costs of a patient with GBS were 267.70 USD (Table 10). Drugs accounted for 69% of total costs, followed by diagnostic tests (17%) and special services (7%). Approximately 84% of the special service costs consisted of specialist consultation costs. Specialist consultations costs consisted mainly of physical therapy and nursing costs. IVIg treatment costs made up 98% of the other treatment costs, because the IVIg treatment was much more expensive (57.26 USD) than treatment with methyl prednisolone (1.29 USD). Large ranges for each cost category indicate costs differed widely between patients. Mean direct non-medical ambulance costs were 26.17 USD per patient (Table 10).

**Table 10:** Direct medical costs and direct non-medical costs in 2020 USD per patient with GBS in a specialised hospital in Gondar, Ethiopia, in 2020

	**Costs/patient (2020 USD)**							**N with costs > 0**
**Cost category**	**Mean**	**St. dev**	**Median**	**Minimum**	**Maximum**	**2.5%**	**97.5%**	
*Direct medical costs*								
Stay costs^a^	11.34	5.12	11.45	1.15	20.61	3.44	20.04	21
Special services costs	18.61	8.19	20.04	5.15	34.07	5.15	32.49	21
Diagnostic test costs	46.52	50.76	13.31	5.73	144.72	6.1	139.71	21
Other treatment costs	5.58	17.41	0	0	58.55	0	57.92	3
Drug costs	185.63	155.43	103.75	7.21	457.77	11.22	436.62	21
Total costs	267.70	185.53	208.06	33.13	590.69	50.19	590.31	21
*Direct non-medical costs*								
Ambulance service costs	26.17	11.68	34.36	0.00	34.36	0.00	34.36	19

^a^Only out-of-pocket costs patients had to pay themselves for each day they stayed in a specialised hospital. These do not cover total costs for a hospital day

### Invasive non-typhoidal salmonellosis

Records of 21 patients with iNTS of a specialised hospital were retrieved. Most patient were male and approximately half came from a rural area (Table 11). At admission, four patients were under five years of age, nine between five and 18 years and eight 18 years and older (mean 15.7 years).

**Table 11:** Sex, area of residence and age of patients with iNTS in a specialised hospital in Gondar, Ethiopia, in 2020

Characteristic	Unit	Answer	Value
Sex	N	Male	17
		Female	4
Area of residence	N	Urban	11
		Rural	9
		Unknown	1
Age at admission	Years	Mean	15.7
		Standard deviation	11.8
		Range	0—45
	N	Under five years	4

Here, we provide a summary of the healthcare resource use of iNTS patients, details can be found in supplementary material S3. All patients left the hospital alive. On average, patients were ill for 3.1 days before entering the hospital (range 0 to 10 days). The type of illness was not recorded. Twenty-eight patients received care before entering the hospital, each from one provider. Most used providers were over-the-counter drugs and a health centre. Patients stayed on average 5.9 days in the hospital, of which 2.6 days in the intensive care unit, 1.8 days in the emergency room, 0.9 days in the paediatric ward and 0.6 days in the outpatient ward. Although an outpatient ward is only for patients that are not hospitalized, several patients stayed a full day or more in the outpatient ward because of a shortage of beds on other wards/units for inpatients. We have no data about the order in which patients stayed in each ward or unit. All patients used specialist consultation, ranging from one to eight times, and a special diet, and almost all used intravenous fluids. All patients used at least one diagnostic test, most only applied once for a patient. Most used tests were full blood count, blood electrolytes, blood glucose and X-ray. All patients received antibiotics and no other drugs. Four different types of antibiotics (azithromycin, ceftriaxone, ciprofloxacin, trimethoprim) were administered.

Mean direct medical costs of a patient with iNTS were 47.79 USD, with a range from 16.61 to 68.49 USD (Table 11). Drugs accounted for 70%, followed by diagnostic tests for 20%. All patients used ambulance service resulting in mean costs of 25.37 USD per iNTS patient (Table 12).

**Table 12:** Direct medical costs and direct non-medical costs in 2020 USD per patient with iNTS in a specialised hospital in Gondar, Ethiopia, in 2020

	**Costs/patient (2020 USD)**							**N with costs > 0**
**Cost category**	**Mean**	**St. dev**	**Median**	**Minimum**	**Maximum**	**2.5%**	**97.5%**	
*Direct medical costs*								
Stay costs^a^	3.35	1.29	2.86	1.72	5.73	1.72	5.73	21
Special services costs	3.38	0.31	3.44	2	3.44	2.72	3.44	21
Diagnostic test costs	8.33	0.86	8.87	6.3	8.87	6.3	8.87	21
Drug costs	32.75	14.69	30.46	1.43	53.31	1.43	52.3	21
Total costs	47.79	13.97	42.20	16.61	68.49	18.04	67.05	21
*Direct non-medical costs*								
Ambulance service costs	25.37	8.79	17.18	17.18	34.36	17.18	34.36	21

^a^Only out-of-pocket costs patients had to pay themselves for each day they stayed in a specialised hospital. These do not cover total costs for a hospital day

The healthcare resource use of patients with iNTS were higher than those of patients with diarrhoea in a specialised hospital. The mean hospital stay of a patient with iNTS (5.9 days) was approximately four times higher than the mean stay of a patient with diarrhoea (1.5 days). Specialist consultation was used for all patients with iNTS, whereas for less than a third of patients with diarrhoea. Special diet was provided to all patients with iNTS, whereas to only 16% of patients with diarrhoea. Intravenous fluids were used similarly for both patients with iNTS and with diarrhoea. More types of drugs and with higher administer frequency and more types of diagnostic tests were used for patients with iNTS than for patients with diarrhoea. The higher healthcare resource use resulted in mean direct medical costs of a patient with iNTS being approximately five times higher than those of a patient with diarrhoea

### National cost-of-illness estimates

The age-weighted probabilities of developing GBS as a sequela to diarrhoea by *Campylobacter* spp. and iNTS to diarrhoea by non-typhoidal *Salmonella enterica* were 0.00042 and 0.13, respectively. Total estimated cost-of-illness in Ethiopia due to FBD related to *Campylobacter* spp., ETEC and non-typhoidal *Salmonella enterica* were 343.4 million USD, of which 55% was related to *Campylobacter* spp., 24% to non-typhoidal *Salmonella enterica* and 21% to ETEC (Table 13). Of the cost-of-illness related to non-typhoidal *Salmonella enterica* of 83.9 million USD, 10.2 million USD (12.2%) was due to iNTS. Of the cost-of-illness related to *Campylobacter* spp. of 187.8 million USD, 0.4 million USD (0.2%) was due to GBS.

**Table 13:** Estimated national cost-of-illness due to foodborne diseases caused by *Campylobacter* spp. (Camp), enterotoxigenic *Escherichia coli* (ETEC) and non-typhoidal *Salmonella enterica* (Salm) in Ethiopia in 2017

Pathogen	Health state	Incidence^a^	Direct medical costs		(In)direct non-medical costs		Total costs per health state (mln USD)	Total costs per pathogen (mln USD)^d^
			**Costs / patient (USD)**^**b**^	**Total (mln**^**c**^** USD)**	**Costs / patient (USD)**^**b**^	**Total (mln USD)**		
Camp	diarrhoea	2.8 mln	8.96	25.1	58	162.4	187.5	187.8
	GBS	1,200	267.70	0.3	26.17	0.0	0.4	
ETEC	diarrhoea	1.1 mln	8.96	9.6	58	62.1	71.6	71.6
Salm	diarrhoea	1.1 mln	8.96	9.9	58	63.8	73.7	83.9
	iNTS	140,000	47.79	6.7	25.37	3.6	10.2	
All				51.5		291.8	343.4	343.4

^a^Diarrhoea incidence from Havelaar, et al. [18]. GBS and iNTS incidence based on age-weighted probabilities of developing GBS and iNTS as sequelae to diarrhoea by *Campylobacter* spp. and non-typhoidal *Salmonella enterica*, respectively, extracted from the FERG database

^b^Diarrhoea from Table 3, GBS from Table 10 and iNTS from Table 12. Note that for GBS and iNTS, these include only ambulance cost whereas for diarrhoea these include transport costs and lost income

^c^million

^d^Differences with sum of costs per health state due to rounding

## Discussion

Our study estimated direct medical, direct non-medical and indirect non-medical costs for patients with diarrhoea and direct medical and direct non-medical costs for patients with GBS and iNTS in Gondar, Ethiopia in 2020. To our knowledge, this is the first study that collected such data about GBS and iNTS in Ethiopia. For diarrhoea, few studies on Ethiopia and other countries on the African continent are available. Per patient with diarrhoea in the specialised hospital, mean direct medical costs were 9.53 USD and in previously visited facilities 13.60 USD, direct non-medical transport costs approximately 15 USD and indirect non-medical costs (lost income) 29.12 USD. Thus, total costs per patient with diarrhoea in the specialised hospital were approximately 67 USD. Direct medical costs in the specialised hospital were thus approximately 15% of the total cost per patient. Total costs in our study are approximately three times higher than the median of direct medical, non-medical and indirect costs in the specialised hospital in the city of Gondar plus costs of treatment before the hospital visit of 22.25 USD per patient coming to the outpatient pharmacy in 2016 [8]. In our study, patients were hospitalised for at least one day, so most likely had more severe illness than patients coming to the outpatient pharmacy, resulting in higher costs. Furthermore, unit costs of healthcare resources could have increase from 2016 to 2020. Our cost estimates for diarrhoea patients in Ethiopia are in the same range as presented in studies in other countries on the African continent. Our total estimated costs of 67 USD are in the range of the mean inpatient total costs related to diarrhoea due to any etiology in children up to 5 years of 11.03 and 151.95 USD per patient for The Gambia, Mali, Mozambique and Kenya in 2012 [4]. Our estimates of the direct medical costs of hospitalisation for diarrhoea patients of 9.53 USD per patient were 5 to 14 times higher than the costs of 0.70 and 2.20 USD per patient estimated in 2011 USD for The Gambia, Kenya and Mali [3], but less than a quarter of the 44.22 USD per patient in Rwanda in 2014 USD [5]. Lost income of 29.12 USD was between 6 and 19 times higher than the 1.55 and 4.99 USD per patient in The Gambia, Kenya or Mali [3], but approximately 70% of the 41.78 USD per patient in Rwanda [5]. The transport costs estimated in our study of approximately 15 USD per patient were in the same order as in Rwanda [5], but higher than the 0.19 to 0.55 USD per patient in The Gambia, Kenya, Mali [3]. It is, however, unclear if the numbers of this last study also include the transportation costs of the patients’ visitors.

For GBS and iNTS, we gathered only data about the direct medical and direct non-medical costs of healthcare resources available on patient records in the specialised hospital. Sum of direct medical costs (267.70 USD) and direct non-medical ambulance costs (26.17 USD) estimated in our study was 293.87 USD per GBS patient and sum of direct medical costs (47.79 USD) and direct non-medical ambulance costs (25.37 USD) was 73.16 USD per iNTS patient. These costs are only part of the total costs related to GBS and iNTS. We did not gather direct medical costs made in previously visited facilities. Direct non-medical costs included only ambulance costs, they did not include transport costs of bringing the patient to the hospital with other means of transport nor the costs of visiting the patient. Furthermore, we did not gather data about the indirect non-medical costs. These do not only include lost income of family caregivers, but potentially also of the patients themselves, because all patients with GBS and eight of the patients with iNTS were 18 years or older. Total costs for a patient with GBS or iNTS can be substantially higher than the costs gathered in our study.

The World Bank estimated gross national income (GNI) per capita in Ethiopia in 2020 at 890 USD (database indicator code NY.GNP.PCAP.CD). Thus, mean total costs of a diarrhoea patient in the specialised hospital of 67 USD were 8% of the GNI per capita. The mean direct medical and direct non-medical ambulance costs per patient with GBS (293.87 USD) and per patient with iNTS (73.16 USD) were 33% and 8% of GNI per capita, respectively. Thus, healthcare costs of a patient with diarrhoea, iNTS or GBS in a specialised hospital can be a considerable part of family income.

On national level, estimated cost-of-illness due to FBD related to *Campylobacter* spp., ETEC and non-typhoidal *Salmonella enterica* were 343.4 million USD. This was 0.4% of the 2017 GNI of 78.379 million USD (The World Bank database indicator code NY.GNP.ATLS.CD), relatively significant in relation to the size of the national economy. Our estimate is lower than the cost-of-illness estimate of 0.6 billion USD related of Jaffee, et al. [2], but we only included the costs due to foodborne disease related to non-typhoidal *Salmonella enterica*, *Campylobacter* spp. and ETEC whereas they included all foodborne diseases. iNTS and GBS contributed 12.2 and 0.2% to the national cost-of-illness related to non-typhoidal *Salmonella enterica* and *Campylobacter* spp., respectively. This suggests that the cost-of-illness related to more severe sequelae need to be included in addition to those related to diarrhoea when assessing the costs related to foodborne hazards causing diarrheal disease in LMIC. The national estimates were based on a relatively small sample in three healthcare facilities in Gondar. Given the skewed distributions of costs, larger sample sizes in more locations across Ethiopia will provide more robust national estimations.

We found few significant differences in costs of diarrhoea patients between *Campylobacter* spp., ETEC and non-typhoidal *Salmonella enterica*. It could be that the mean costs of a diarrhoea patient in the cost categories and healthcare facilities for which we did not find significant differences between the pathogens are similar. However, for part of these costs, especially those for each specific healthcare facility, we might not have found significant differences because of small sample sizes. Furthermore, we don’t know the accuracy of the etiology data collected from patients’ records. For example, many red blood cells and pus cells were recorded as etiology for diarrhoea of several patients (Supplementary material S3). However, this is unlikely to be the cause for diarrhoea but more a result of an infection. This could be due to inaccurate data recording, potentially because physicians take more care and attention in clearly writing prescriptions than in writing test results on lab result sheets. If a lab result sheet is unreadable or lost, nurses or healthcare workers transferring the results to the patient’s record could record whatever they feel appropriate.

In this study, we categorized the costs according to direct and indirect, and medical and non-medical costs. Following standard practice [9, 14], we included direct medical costs, direct non-medical costs and indirect non-medical costs. Indirect medical costs were not included. Other categorizations of costs are possible, for example in costs within the healthcare system, costs of patients and family members, and costs in other sectors [19]. The costs estimated in our study can be rearranged in other categories using the details provided in this manuscript.

In many LMICs, major gaps exist in primary care facilities and hospitals in availability of essential diagnostic tests, such as basic chemistry tests, automatic complete blood count, gram stain, x-ray and ultrasound [20]. This could also be the case in the healthcare facilities in Ethiopia in which we collected data. Thus, the diagnostic tests we retrieved from patient records might differ from the ideal diagnostic tests used in high-income countries (HICs) for such patients. For GBS, insufficient diagnostic and healthcare facilities in many LMICs contribute to delayed diagnosis in patients and a lack of national clinical guidelines and absence of affordable, effective treatments contribute to worse outcomes and higher mortality in LMICs than HICs [21]. Improved diagnostics, health care facilities, guidelines and treatments could lower the health impact of diseases related to FBD, such as diarrhoea, GBS and iNTS. However, the best globally available treatments might, in practice, not be available for patients, because it is too expensive for patients due to their low income and lack of coverage by the health insurance system [21]. Furthermore, the time for a patient to reach a healthcare facility might be (too) long, staff might not be sufficiently skilled to appropriately use equipment even if available, or preferences from provider or patient might prevent appropriate use of available diagnostic services [20].

Our study presented patient-related costs for diarrhoea, GBS and iNTS retrieved from existing registration systems in the healthcare facilities and from interviews with a small sample of family caregivers. These costs present only part of the total healthcare costs in Ethiopia. For example, it excludes direct medical costs that are paid for by direct funding of the healthcare system of the government, health insurance or other organizations, and costs in other types of public and private healthcare facilities and in other regions in Ethiopia. Thus, the cost estimates for diarrhoea, GBS and iNTS provided in our study are a lower limit. Further efforts to quantify such other costs will complement our estimates. We recommend the Ethiopian government, governments in other LMICs and organisations interested in improving food safety in LMICs to increase efforts to gather data about healthcare resource use and associated costs related to FBD, including those for more severe sequalae of foodborne hazards causing diarrheal disease. For this, it is important that healthcare facilities accurately register patients healthcare use. With such data, estimates of the economic burden associated with FBD can be improved, informing decision makers in Ethiopia and other LMICs better about the size of the problem and motivating them more to take action to mitigate risks and prevent illness.

## Conclusion

Mean patient-related direct medical costs in a specialised hospital in Gondar of a patient with GBS (267.70 USD) and with iNTS (47.79 USD) were 30 respectively five times those of a diarrhoea patient (8.96 USD). Differences in mean total direct medical costs of diarrhoea patients were mainly associated with the healthcare facility and not with etiology (*Campylobacter* spp., ETEC, non-typhoidal *Salmonella enterica*). Mean direct medical plus (in)direct non-medical costs per patient with diarrhoea, GBS and iNTS were 8%, 33% and 8% of the GNI per capita, respectively. These costs can be a substantial part of a household’s income. iNTS and GBS contributed 12.2 and 0.2% to the estimated national cost-of-illness related to non-typhoidal *Salmonella enterica* and *Campylobacter* spp., respectively. More severe sequalae can add substantially to cost-of-illness of foodborne hazards causing diarrheal disease. Larger sample sizes in more locations across Ethiopia will provide more robust estimates and further efforts to quantify e.g., costs borne by health care insurance and government will complement these estimates.

## Supplementary Information

**Additional file 1. Supplementary material S1.** Data gathering tools.

**Additional file 2. Supplementary material S2.** Unit costs of healthcare resource use.

**Additional file 3. Supplementary material S3.** Healthcare resource use from patient records. 

**Additional file 4. Supplementary material S4.** Median direct medical costs per diarrhoeapatient by etiology and healthcare facility.

## Data Availability

The datasets used and/or analysed during the current study are available from the corresponding author on reasonable request.

## Abbreviations

ETB: Ethiopian Birr
ETEC: enterotoxigenic *Escherichia coli*
FBD: Foodborne disease
FERG: Foodborne Disease Burden Epidemiology Reference Group
GBS: Guillain-Barré syndrome
GNI: gross national income
HICs: High-income countries
iNTS: invasive non-typhoidal salmonellosis
IVIg: intravenous immunoglobulin
LMICs: low- and middle-income countries
ORS: oral rehydration solution
USD: United States Dollar
WHO: World Health Organization.
