# Supplementary material for: Patient-related healthcare costs for diarrhoea, Guillain Barré syndrome and invasive non-typhoidal salmonellosis in Gondar, Ethiopia, 2020
Source: BMC Public Health. 2022 Nov 16;22:2091. doi: 10.1186/s12889-022-14539-1 (PMC9670532; doi:10.1186/s12889-022-14539-1)
Supplement: Supplementary file 1 — Additional file 1. Supplementary material S1. Data gathering tools. [file 12889_2022_14539_MOESM1_ESM.docx]

**Supplementary material to Direct and indirect (non)-medical patient-related healthcare costs for diarrhoea, GBS, and iNTS in three healthcare facilities in Gondar, Ethiopia in 2020**

**Data gathering tools**

## Diarrhoea: patient records

**Facility information**

1. Type of facility:
2. Specialised hospital
3. Public health centre
4. Private clinic
5. Facility name (code) _______________________________________________________

**Patient information**

1. Patient study ID ______________
2. Patient ID No. from hospital record ________________________
3. Date of admission day _______ month _______ year _________
4. Date of discharge day _______ month _______ year _________
5. Patient age in: days _______ months _________ years _______
6. Patient area of residence:
7. Urban
8. Rural
9. Unknown
10. Height ______.____ cm Weight _______.____ kg Missing/not applicable □
11. Sex of patient:
12. Male
13. Female
14. Missing

**Patient treatment/Diagnostic history**

1. Number of days of diarrhoea before visiting this facility _________________
2. Did the patient receive any care before arriving to this facility?
3. Yes *(continue with question 13)*
4. No *(continue with question 14)*
5. Where did the patient receive care prior to arriving to this facility? (*Multiple responses allowed*).
6. Not applicable
7. Traditional healer
8. Herbalist
9. Over-the-counter drugs
10. Another facility similar to this
11. Health facility/health post
12. Health centre
13. Primary hospital
14. Referral hospital
15. Private health institution
16. Others, specify _______________________________________________________
17. Outcome on discharge from this facility
18. Alive, well
19. Alive, partially recovered
20. Died
21. Alive, but outcome unknown/missing
22. Referred
23. Discharged against medical advice
24. Absconded
25. Missing/unknown
26. What is the admission diagnosis? ___________________________________________
27. What is the final diagnosis?
28. Diarrhoea with no dehydration
29. Diarrhoea with some dehydration
30. Diarrhoea with severe dehydration
31. How many co-morbidities did the patient have? ____________________
32. Specify the co-morbidities _________________________________________________________________
33. Medication use (include any drugs prescribed on discharge). *Write “missing” for any data not there (e.g. if number of days administered is missing)*.

| **Name of drug** | **Route*** | **Dose units** | **Dose amount** | **Frequency of administering** | **No. of days administered during admission** | **No. of days administered after discharge** |
| --- | --- | --- | --- | --- | --- | --- |
|  |  | **(e.g. μg/ml, ml, mg)** | **(e.g. 50, 100)** | **(e.g. once/day, 3/ day)** | **(e.g. 3 days)** | **(e.g. 3 days)** |
|  |  |  |  |  |  |  |
|  |  |  |  |  |  |  |

* 1 = intravenous; 2 = injection; 3 = oral; 4 = nasal/gastric tube; 5 = rectal; 6 = topical (ointments); 7 = drops for ear, nose, throat; 8 = inhalation

1. Were any of these diagnostic tests used? (*If “Yes” but no quantity stated, write “missing” in last column.*)

| **Type** | **Circle** | **Number of tests performed** |
| --- | --- | --- |
| Blood test | 1. No 2. Yes |  |
| Blood culture | 1. No 2. Yes |  |
| Urinalysis | 1. No 2. Yes |  |
| Stool | 1. No 2. Yes |  |
| Other (specify):_____________ |  |  |

1. What was the cause of the diarrhoeal illness?
2. Non-typhoidal *Salmonella enterica*
3. *Campylobacter* spp.
4. Enterotoxigenic *Escherichia coli* (ETEC)
5. Other, specify _________________________________________________________
6. Missing
7. Length of stay by location (record length of stay in each type of room, including zeros). *(If length of stay is 1–11 hours write ½ day. If 12–24 hours, write 1 day.)*

| **Type of room** | **Length of stay (days)** |
| --- | --- |
| Outpatient clinic |  |
| Paediatric ward |  |
| Intensive care unit or special care baby unit |  |
| Isolation unit |  |
| Emergency room |  |
| Other (specify) _____________________________ |  |
| Missing |  |

1. Were any of these other special services used? *(If “Yes” but no quantity stated, write “missing” in last column.)*

| **Type** |  | **Unit of measure where applicable** | **Quantity** |
| --- | --- | --- | --- |
| Ambulance services | 1. No 2. Yes |  |  |
| Special diet | 1. No 2. Yes |  |  |
| Specialist consultations | 1. No 2. Yes |  |  |
| Intravenous fluids | 1. No 2. Yes |  |  |
| Cardiopulmonary resuscitation | 1. No 2. Yes |  |  |
| Autopsy | 1. No 2. Yes |  |  |
| Other (specify):_____________ |  |  |  |

## Diarrhoea: family caregiver interviews

**Facility information**

1. Type of facility:
2. Specialised hospital
3. Public health centre
4. Private clinic
5. Facility name (code): _____________________________________________________

**Patient information:**

1. Patient study ID __________________________________________________________
2. Patient ID from patient records: ______________________________________________
3. Gender
4. Male
5. Female
6. Missing
7. Patient age in: days _______ months _________ years _______
8. Patient area of residence:
9. Urban
10. Rural
11. Unknown
12. Informed consent date: day ______ month ______ year ________

**Caregiver information:**

1. Relationship to the patient:
2. Mother
3. Father
4. Sister
5. Brother
6. Grandmother
7. Grandfather
8. Other relative
9. Friend
10. Other (specify): ____________________________________

**Travel information**

1. How long did it take to get here from your home (including the journey time and any waiting for transport)? *(tick unknown if no time is mentioned)*

Hours ________ Minutes _________ Unknown □

1. What kind of transportation did you use to bring the patient to this hospital or clinic? *(In case of multiple means of transportation during this trip, please tick only the transportation that was used for the longest distance.)*
2. Car
3. Bus / train
4. Bicycle
5. Motorbike
6. Taxi
7. By foot
8. Boat
9. Ambulance
10. Other, specify: ________________________________________________________
11. If you paid for transportation to bring the patient to the hospital or clinic, how much did you pay? __________ *(put 0 if no payment was made)*
12. How many trips did you or other household members make to visit the patient? *(Total numbers of round trips)* ____________________ *(put* ***0*** *if no visit was made)*

*Examples: 3 relatives’ visit one time [****n = 3 trips****]; One relative visits three times [****n = 3 trips****]*

1. What kind of transportation did you use to come to this hospital or clinic to visit the patient? *(It concerns the last used transportation that has been used to visit the patient.)*
2. Car
3. Bus / train
4. Bicycle
5. Motorbike
6. Taxi
7. By foot
8. Boat
9. Other, specify ________________________________________________________
10. If you paid for transportation, how much did you pay to visit this healthcare facility? *(for one round trip and one person) ____________________________ (Put* ***0*** *if no payment was made and 999 if don’t know). If you used different means of transportation, please choose the one you used most often.*
11. Treatment costs

Before visiting this facility, did you seek help from any of the following? How much did it cost you for drugs, tests, consultation and other financial costs? (*Caregiver to list all the facilities visited, then ask the costs of each item for each place visited, one at a time.)*

| **Facility Expenditure** | **Private hospital** | **Public clinic** | **Private clinic** | **Pharmacy** | **Traditional healer** | **Friend** | **Shop** | **Other** |
| --- | --- | --- | --- | --- | --- | --- | --- | --- |
| Drug |  |  |  |  |  |  |  |  |
| Diagnostic / tests |  |  |  |  |  |  |  |  |
| Consultation |  |  |  |  |  |  |  |  |
| Other financial costs |  |  |  |  |  |  |  |  |
| Total costs |  |  |  |  |  |  |  |  |

1. How much did the household actually pay for: drugs, tests, consultation and other fees for this visit or hospitalization? How much were you required to pay?

| **Item** | **Drug** | **Tests** | **Consultation fee** | **Other fees** | **Total** |
| --- | --- | --- | --- | --- | --- |
| Cost *(Put 0 if no payment and 999 if don’t know)* |  |  |  |  |  |
| How much were you required to pay? |  |  |  |  |  |

1. Are you losing some income for being here today?
2. Yes
3. No
4. If you weren’t here today, what would you be doing? *(Multiple responses allowed.)*
5. Nothing
6. Housework
7. Looking after my children
8. Working (specify)
9. Other (specify)
10. Don’t know
11. How much income (in total) have you and other family members lost as a result of taking care of the patient instead of working? *(Put 0 if nothing and 999 if don’t know.)* __________________________________________________

**Financing of the costs of treatment and transport**

1. Has the illness affected the family financially?
2. Yes
3. No
4. Where did the money come from to pay for these expenses? *(Multiple responses allowed.)*
5. Cutting down on other expenses
6. Using savings
7. Borrowing
8. Selling assets
9. Asking for donations from friends and relatives
10. Others, specify _______________________________________________________
11. Date of admission day _______ month _______ year _________

Date of discharge / referral day _______ month _______ year _________

## Guillain-Barré Syndrome: patient records

**Facility information**

1. Type of facility:
2. Specialised hospital
3. Public health centre
4. Private clinic
5. Facility name (code): _____________________________________________________

**Patient information**

1. Patient study ID ________________________
2. Patient ID No. from hospital record ______________________________
3. Date of admission day _______ month _______ year _________
4. Date of discharge day _______ month _______ year _________
5. Patient age in: days _______ months _________ years _______
6. Patient area of residence:
7. Urban
8. Rural
9. Unknown
10. Height ______.____ cm Weight _______.____ kg Missing/not applicable □
11. Sex of patient:
12. Male
13. Female
14. Missing

**Patient treatment/Diagnostic history**

1. Number of days of illness before hospitalization ________________________
2. Did the patient receive any care before arriving to this facility?
3. Yes *(continue with question 13)*
4. No *(continue with question 14)*
5. Where did the patient receive care prior to arriving to this facility? *(Multiple responses allowed).*
6. Not applicable
7. Traditional healer
8. Herbalist
9. Over-the-counter drugs
10. Another facility similar to this
11. Health facility/health post
12. Health center
13. Primary hospital
14. Referral hospital
15. Private health institution
16. Others, specify _______________________________________________________
17. Outcome on discharge from this facility
18. Alive, well
19. Alive, partially recovered
20. Died
21. Alive, but outcome unknown/missing
22. Referred
23. Discharged against medical advice
24. Absconded
25. Missing/unknown
26. What is the admission diagnosis? ___________________________________________
27. What is the final diagnosis?
28. Mild GBS
29. Severe GBS
30. How many co-morbidities did the patient have? ____________________
31. Specify the co-morbidities _________________________________________________________________
32. Length of stay by location (record length of stay in each type of room, including zeros). *(If length of stay is 1–11 hours write ½ day. If 12–24 hours, write 1 day.)*

| **Type of room** | **Length of stay (days)** |
| --- | --- |
| Outpatient clinic |  |
| Paediatric ward |  |
| Intensive care unit or special care baby unit |  |
| Isolation unit |  |
| Emergency room |  |
| Other (specify) _____________________________ |  |
| Missing |  |

1. Were any of these other special services used? *(If “Yes” but no quantity stated, write “missing” in last column.)*

| **Type** |  | **Unit of measure where applicable** | **Quantity** |
| --- | --- | --- | --- |
| Ambulance services | 1. No 2. Yes |  |  |
| Special diet | 1. No 2. Yes |  |  |
| Specialist consultations | 1. No 2. Yes |  |  |
| Intravenous fluids | 1. No 2. Yes |  |  |
| Cardiopulmonary resuscitation | 1. No 2. Yes |  |  |
| Autopsy | 1. No 2. Yes |  |  |
| Other (specify): ____________ |  |  |  |

1. Which diagnostic tests were used? (Tick the applicable tests)
2. **No diagnostic test**
3. **Blood count. (***If “yes”, which one(s)***)**
   1. Full blood count/complete blood count _______________________________
   2. Hb/haemoglobin ________________________________________________
   3. Haematocrit/packed cell volume (HCT/PCV) __________________________
4. **Blood culture**
5. **HIV test**
   1. ELISA ________________________________________________________
   2. Rapid test _____________________________________________________
6. **Other microbiology (***If “yes”, which one(s)and how many***)**
   1. Name(s) of sample ______________________________________________
   2. Gram stain/microscopy ___________________________________________
   3. Culture ________________________________________________________
   4. Sensitivity _____________________________________________________
   5. Antigen testing
7. **Radiology (***If “yes”, which one(s)***)**
   1. Computed tomography (CT scan) ___________________________________
   2. Ultrasound _____________________________________________________
   3. Other X-ray ____________________________________________________
8. **Blood chemistry (***If “yes”, which one(s)***)**
   1. Electrolytes ____________________________________________________
   2. Glucose _______________________________________________________
9. **Spinal puncture**
10. **EMG examination**
11. **Other (specify) _______________________________________________________**
12. What further treatment was applied to the patient and in what amounts?

| **Treatment** | **Number of treatments** |
| --- | --- |
| IVIg treatment |  |
| Methyl prednisolon (MP) treatment |  |
|  |  |

1. How many consultations/sessions did the patient have with a specialist during the stay in the hospital?

| **specialist** | **Number of consultations/sessions with this specialist** |
| --- | --- |
| Physiotherapist |  |
| Neurologist |  |
| Rehabilitation doctor |  |
| Other, specify____________ |  |

1. Drugs taken (include any drugs prescribed on discharge). *Write “missing” for any data not there (e.g. if number of days administered is missing).*

| **Name of drug** | **Route*** | **Dose units** | **Dose amount** | **Frequency of admini-stering** | **No. of days administered during admission** | **No. of days administered after discharge** |
| --- | --- | --- | --- | --- | --- | --- |
|  |  | **(e.g. μg/ml, ml, mg)** | **(e.g. 50, 100)** | **(e.g. once/day, 3/ day)** | **(e.g. 3 days)** | **(e.g. 3 days)** |
|  |  |  |  |  |  |  |
|  |  |  |  |  |  |  |

* 1 = intravenous; 2 = injection; 3 = oral; 4 = nasal/gastric tube; 5 = rectal; 6 = topical (ointments); 7 = drops for ear, nose, throat; 8 = inhalation

1. Please describe any unusual or complicated case that you come across.

____________________________________________________________________________________________________________________________________________________________________________________

## Invasive non-typhoidal salmonellosis: patient records

**Facility information**

1. Type of facility:
2. Specialised hospital
3. Public health centre
4. Private clinic
5. Facility name (code): ______________________________________________________

**Patient information**

1. Patient study ID _______________________
2. Patient ID No. from hospital record ____________________________
3. Date of admission day _______ month _______ year _________
4. Date of discharge day _______ month _______ year _________
5. Patient age in: days _______ months _________ years _______
6. Patient area of residence:
7. Urban
8. Rural
9. Unknown
10. Height ______.____ cm Weight _______.____ kg Missing/not applicable □
11. Sex of patient:
12. Male
13. Female
14. Missing

**Patient treatment/Diagnostic history**

1. Number of days of illness before hospitalization ______________________
2. Did the patient receive any care before arriving to this facility?
3. Yes *(continue with question 13)*
4. No *(continue with question 14)*
5. Where did the patient receive care prior to arriving to this facility? *(Multiple responses allowed).*
6. Not applicable
7. Traditional healer
8. Herbalist
9. Over-the-counter drugs
10. Another facility similar to this
11. Health facility/health post
12. Health center
13. Primary hospital
14. Referral hospital
15. Private health institution
16. Others, specify _______________________________________________________
17. Outcome on discharge
18. Alive, well
19. Alive, partially recovered
20. Died
21. Alive, but outcome unknown/missing
22. Referred
23. Discharged against medical advice
24. Absconded
25. Missing/unknown
26. What is the admission diagnosis? ___________________________________________
27. What is the final diagnosis? ________________________________________________
28. How many co-morbidities did the patient have? ________________________
29. Specify the co-morbidities _________________________________________________________________
30. Length of stay by location (record length of stay in each type of room, including zeros). *(If length of stay is 1–11 hours write ½ day. If 12–24 hours, write 1 day.)*

| **Type of room** | **Length of stay (days)** |
| --- | --- |
| Outpatient clinic |  |
| Paediatric ward |  |
| Intensive care unit or special care baby unit |  |
| Isolation unit |  |
| Emergency room |  |
| Other (specify) _____________________________ |  |
| Missing |  |

1. Were any of these other special services used? *(If “Yes” but no quantity stated, write “missing” in last column.)*

| **Type** |  | **Unit of measure where applicable** | **Quantity** |
| --- | --- | --- | --- |
| Ambulance services | 1. No 2. Yes |  |  |
| Special diet | 1. No 2. Yes |  |  |
| Specialist consultations | 1. No 2. Yes |  |  |
| Intravenous fluids | 1. No 2. Yes |  |  |
| Cardiopulmonary resuscitation | 1. No 2. Yes |  |  |
| Autopsy | 1. No 2. Yes |  |  |
| Other (specify): _____________ |  |  |  |

1. Which diagnostic tests were used? *(Tick the applicable tests)*
2. **No diagnostic test**
3. **Blood count. (***If “yes”, which one(s)***)**
   1. Full blood count/complete blood count _______________________________
   2. Hb/haemoglobin ________________________________________________
   3. Haematocrit/packed cell volume (HCT/PCV) __________________________
4. **Blood culture**
5. **Bone marrow cultures**
6. **Rapid serology**
   1. Widal agglutination test
   2. Tubex TF
   3. Typhidot TF ELISA
   4. RTI
7. **Antigen**
8. **Polymerase Chain Reaction (PCR)**
9. **HIV test**
   1. ELISA ________________________________________________________
   2. Rapid test _____________________________________________________
10. **Other microbiology (***If “yes”, which one(s)and how many***)**
    1. Name(s) of sample ______________________________________________
    2. Gram stain/microscopy ___________________________________________
    3. Culture ________________________________________________________
    4. Sensitivity _____________________________________________________
    5. Antigen testing
11. **Radiology (***If “yes”, which one(s)***)**
    1. Computed tomography (CT scan) ___________________________________
    2. Ultrasound _____________________________________________________
    3. Other X-ray ____________________________________________________
12. **Blood chemistry (***If “yes”, which one(s)***)**
    1. Electrolytes ____________________________________________________
    2. Glucose _______________________________________________________
13. **Other (specify) _______________________________________________________**
14. Drugs taken *(include any drugs prescribed on discharge). Write “missing” for any data not there (e.g. if number of days administered is missing).*

| **Name of drug** | **Route*** | **Dose units** | **Dose amount** | **Frequency of admini-stering** | **No. of days administered during admission** | **No. of days administered after discharge** |
| --- | --- | --- | --- | --- | --- | --- |
|  |  | **(e.g. μg/ml, ml, mg)** | **(e.g. 50, 100)** | **(e.g. once/day, 3/ day)** | **(e.g. 3 days)** | **(e.g. 3 days)** |
|  |  |  |  |  |  |  |
|  |  |  |  |  |  |  |

* 1 = intravenous; 2 = injection; 3 = oral; 4 = nasal/gastric tube; 5 = rectal; 6 = topical (ointments); 7 = drops for ear, nose, throat; 8 = inhalation

1. Please describe any unusual or complicated case that you come across.

____________________________________________________________________________________________________________________________________________________________________________________.
