# Supplementary material for: Patient-related healthcare costs for diarrhoea, Guillain Barré syndrome and invasive non-typhoidal salmonellosis in Gondar, Ethiopia, 2020
Source: BMC Public Health. 2022 Nov 16;22:2091. doi: 10.1186/s12889-022-14539-1 (PMC9670532; doi:10.1186/s12889-022-14539-1)
Supplement: Supplementary file 2 — Additional file 2. Supplementary material S2. Unit costs of healthcare resource use. [file 12889_2022_14539_MOESM2_ESM.docx]

**Supplementary material to Direct and indirect (non)-medical patient-related healthcare costs for diarrhoea, GBS, and iNTS in three healthcare facilities in Gondar, Ethiopia, in 2020**

**Unit costs of healthcare resource use**

# Diarrhoea

Table 1 gives the unit costs of a hospital day, special services and diagnostic tests used for diarrhoea patients in each of the three healthcare facilities. Table 2 gives the unit costs for the medication used. Table 3 gives the input data to estimate the ambulance service costs.

**Table 1: Healthcare resource costs excluding medications.**

| **Healthcare resource** | **Health centre** | | **Private clinic** | | **Specialised hospital** | | **Unit** |
| --- | --- | --- | --- | --- | --- | --- | --- |
|  | **ETB** | **USD ^1^** | **ETB** | **USD ^1^** | **ETB** | **USD ^1^** |  |
| *Healthcare facility stay* |  |  |  |  |  |  |  |
| Costs per day (outpatient, pediatric, intensive care, emergency room, antenatal care)^2^ | 15 | 0.43 | 50 | 1.43 | 20 | 0.57 | /day |
| *Special services* |  |  |  |  |  |  |  |
| Special diet | 30 | 0.86 | 80 | 2.29 | 40 | 1.15 | /diet |
| Specialist consultation | 20 | 0.57 | 80 | 2.29 | 30 | 0.86 | /consult |
| Intravenous fluids | 40 | 1.15 | 70 | 2.00 | 50 | 1.43 | /bag |
| Cardiopulmonary resuscitation (CPR) | 50 | 1.43 | 70 | 2.00 | 60 | 1.72 | /CPR |
| *Diagnostic tests* |  |  |  |  |  |  |  |
| Blood test | 20 | 0.57 | 40 | 1.15 | 30 | 0.86 | /blood test |
| Blood culture | 20 | 0.57 | 40 | 1.15 | 30 | 0.86 | /blood culture |
| Urine test | 25 | 0.72 | 45 | 1.29 | 40 | 1.15 | /urine test |
| Stool test | 25 | 0.72 | 45 | 1.29 | 40 | 1.15 | /stool test |

^1^ Using 2020 average official exchange rate of 34.93 ETB per USD (The World Bank, indicator code PA.NUS.FCRF).^2^ Only out-of-pocket costs patients had to pay themselves for each day they stayed in the healthcare facility. These do not cover total costs for one hospital day.

**Table 2: Unit costs of medication used (same for all healthcare facilities).**

| **Medicine** | **Route** | **Size of unit** | **Dose unit** | **price per unit** | |
| --- | --- | --- | --- | --- | --- |
|  |  |  |  | **ETB** | **USD ^1^** |
| Albendazole | Oral | 200 | mg | 15 | 0.43 |
| Albendazole | Oral | 400 | mg | 25 | 0.72 |
| Amoxicillin | Oral | 250 | mg | 2 | 0.06 |
| Amoxicillin | Oral | 500 | mg | 3 | 0.09 |
| Ampicillin | Intravenous | 250 | mg | 10 | 0.29 |
| Ampicillin | Intravenous | 500 | mg | 15 | 0.43 |
| Coartem | Oral | 20 | mg | 50 | 1.43 |
| Ceftriaxone | Intravenous | 500 | mg | 9 | 0.26 |
| Ceftriaxone | Intravenous | 1000 | mg | 15 | 0.43 |
| Ciprofloxacin | Oral | 500 | mg | 9 | 0.26 |
| Cloxacillin | Intravenous | 250 | mg | 4 | 0.11 |
| Cloxacillin | Intravenous | 500 | mg | 10 | 0.29 |
| Cotrimoxazole | Oral | 200 | mg | 2 | 0.06 |
| Dexamethasone | Intravenous | 4 | mg | 4 | 0.11 |
| Diclofenac | Injection | 25 | mg | 3 | 0.09 |
| Gentamycin | Intravenous | 80 | mg | 4 | 0.11 |
| Glucose 40% | Intravenous | 20 | ml | 6 | 0.17 |
| Ringer’s solution | Intravenous | 500 | ml | 20 | 0.57 |
| Mebendazole | Oral | 100 | mg | 2 | 0.06 |
| Metoclopramide | Oral | 5 | mg | 15 | 0.43 |
| Metoclopramide | Oral | 100 | ml | 40 | 1.15 |
| Metronidazole | Oral | 250 | mg | 2 | 0.06 |
| Normal saline | Intravenous | 500 | ml | 20 | 0.57 |
| Normal saline | Intravenous | 1000 | ml | 30 | 0.86 |
| Omeprazole | Oral | 20 | mg | 2 | 0.06 |
| ORS | Oral/Nasal/gastric tube | 1 | l | 10 | 0.29 |
| Paracetamol | Oral | 100 | mg | 3 | 0.09 |
| Paracetamol | Oral | 500 | mg | 5 | 0.14 |
| Paracetamol | Rectal | 125 | mg | 5 | 0.14 |
| Praziquantel | Oral | 600 | mg | 10 | 0.29 |
| Protec | Injection | 100 | mg | 132 | 3.78 |
| Protec | Oral | 100 | mg | 30 | 0.86 |
| Vitamin B complex | Intravenous | 5 | ampule | 5 | 0.14 |
| Zinc tablet | Oral | 20 | mg | 10 | 0.29 |

^1^ Using 2020 average official exchange rate of 34.93 ETB per USD (The World Bank, indicator code PA.NUS.FCRF).

**Table 3: Input data used to estimate ambulance service costs per healthcare facility.**

| **Healthcare resource** | **Health centre** | **Private clinic** | **Specialised hospital** | **Unit** |
| --- | --- | --- | --- | --- |
| Ambulance service costs | 30 | 70 | 30 | ETB/km |
|  | 0.86 | 2.00 | 0.86 | USD/km ^1^ |
| Estimated distance from urban area | 25 | 20 | 20 | km |
| Estimated distance from rural area | 50 | 40 | 40 | km |

^1^ Using the 2020 average official exchange rate of 34.93 ETB per USD (The World Bank, indicator code PA.NUS.FCRF).

# Guillain-Barré syndrome

Table 4 gives the unit costs of a hospital day, special services and diagnostic tests used for patients with GBS in a specialized hospital in Gondar, Ethiopia. Table 5 gives the unit costs for the medication used in the hospital. Table 6 gives the input data to estimate the ambulance service costs.

**Table 4: Data used to estimate the healthcare resource costs of the GBS patients in a specialised hospital.**

| **Healthcare resource** | **Price** | | **Unit** |
| --- | --- | --- | --- |
|  | **ETB** | **USD ^1^** |  |
| *Healthcare facility stay* |  |  |  |
| Costs per day ^1^ | 20 | 0.57 | /day |
| *Special services* |  |  |  |
| Special diet | 40 | 1.15 | /diet |
| Intravenous fluids | 50 | 1.43 | /bag |
| Cardiopulmonary resuscitation | 60 | 1.72 | /cardiopulmonary resuscitation |
| *Diagnostic tests* |  |  |  |
| Blood count: full count | 30 | 0.86 | /full count test |
| Blood count: Hb | 30 | 0.86 | /HB test |
| Blood count: HCT/PCV | 30 | 0.86 | /HCT/PCV test |
| Blood culture | 30 | 0.86 | /blood culture test |
| HIV: rapid test | 30 | 0.86 | /HIV rapid test |
| Microbiology: gram stain/microscopy | 15 | 0.43 | / gram stain/microscopy test |
| Microbiology: culture | 30 | 0.86 | /culture test |
| Microbiology: sensitivity | 65 | 1.86 | /sensitivity test |
| Microbiology: antigen | 65 | 1.86 | /antigen test |
| Radiology: CT scan | 1800 | 51.54 | /CT scan |
| Radiology: ultrasound | 60 | 1.72 | /ultrasound |
| Radiology: X-ray | 120 | 3.44 | /X-ray |
| Blood chemistry: electrolytes | 30 | 0.86 | /electrolyte test |
| Blood chemistry: glucose | 15 | 0.43 | /glucose test |
| Other test: MRI | 2500 | 71.58 | /MRI |
| Other test: ECG | 50 | 1.43 | /ECG |
| *Other treatments* |  |  |  |
| IVIg treatment | 2000 | 57.26 | /treatment |
| Methyl prednisolone treatment | 45 | 1.29 | /treatment |
| *Specialist consultation* |  |  |  |
| Physiotherapist | 20 | 0.57 | /consult |
| Neurologist | 30 | 0.86 | /consult |
| Nurse | 20 | 0.57 | /consult |

^1^ Using 2020 average official exchange rate of 34.93 ETB per USD (The World Bank, indicator code PA.NUS.FCRF). ^2^ Only out-of-pocket costs patients had to pay themselves for each day they stayed in the healthcare facility. These do not cover total costs for one hospital day.

**Table 5: Unit costs of medication used for patients with GBS in a specialised hospital.**

| **Drug** | **Route** | **Dose amount** | **Dose unit** | **Price per unit** | |  | **Unit amount** | **Dose unit** |
| --- | --- | --- | --- | --- | --- | --- | --- | --- |
|  |  |  |  | **ETB** | **USD^1^** |  |  |  |
| Acyclovir | intravenous | 500 | mg | 80 | 2.29 | /vial | 500 | mg |
| Acyclovir | oral | 300 | mg | 80 | 2.29 | /vial | 200 | mg |
| Acyclovir | oral | 500 | mg | 80 | 2.29 | /vial | 200 | mg |
| Amitriptyline | oral | 12.5 | mg | 30 | 0.86 | cent/tab | 10 | mg |
| Azithromycin | oral | 500 | mg | 20 | 0.57 | /tab | 250 | mg |
| Bisacodyl | oral | 5 | mg | 50 | 1.43 | cent/tab | 5 | mg |
| Cefepime | intravenous | 2 | mg | 132 | 3.78 | /tab | 1 | mg |
| Ceftazidime | intravenous | 2 | g | 75 | 2.15 | /vial | 0.5 | g |
| Ceftriaxone | Intravenous | 1 | g | 9 | 0.26 | /vial | 0.25 | g |
| Ceftriaxone | Intravenous | 2 | g | 9 | 0.26 | /vial | 0.25 | g |
| Dexamethasone | intravenous | 4 | mg | 4 | 0.11 | /vial | 4 | mg |
| Dexamethasone | intravenous | 8 | mg | 4 | 0.11 | /vial | 4 | mg |
| Dexamethasone | intravenous | 12 | mg | 4 | 0.11 | /vial | 4 | mg |
| Diclofenac | oral | 50 | mg | 3 | 0.09 | /vial | 25 | mg |
| Enalapril | oral | 2.5 | mg | 0.5 | 0.01 | /tab | 2.5 | mg |
| Lactulose | oral | 45 | ml | 150 | 4.29 | /bottle | 15 | ml |
| Lasix | intravenous | 80 | mg | 3 | 0.09 | /vial | 10 | mg |
| Metronidazole | intravenous | 500 | mg | 7 | 0.20 | /bottle | 500 | mg |
| Metronidazole | oral | 500 | mg | 7 | 0.20 | /bottle | 250 | mg |
| Omeprazole | oral | 20 | mg | 45 | 1.29 | /vial | 10 | mg |
| Potassium chloride | intravenous | 40 | mmol | 20 | 0.57 | /vial | 10 | mmol |
| Prednisolone | oral | 50 | mg | 0.5 | 0.01 | /tab | 5 | mg |
| Tramadol | intravenous | 50 | mg | 6 | 0.17 | /vial | 50 | mg |
| UFH | Subcutaneous | 5000 | IU | 104 | 2.98 | /vial | 1000 | IU |
| Vancomycin | intravenous | 250 | mg | 45 | 1.29 | /vial | 500 | mg |
| Vancomycin | intravenous | 1 | g | 45 | 1.29 | /vial | 500 | mg |

^1^ Using 2020 average official exchange rate of 34.93 ETB per USD (The World Bank, indicator code PA.NUS.FCRF).

**Table 6: Data used to estimate the direct non-medical healthcare resource costs of the GBS patients in a specialised hospital.**

|  | **price** | **unit** |
| --- | --- | --- |
| Ambulance service | 30 | ETB/km |
|  | 0.86 | USD/km ^1^ |
| Urban | 20 | km |
| Rural | 40 | km |

^1^ Using the 2020 average official exchange rate of 34.93 ETB per USD (The World Bank, indicator code PA.NUS.FCRF).

# Invasive non-typhoidal salmonellosis

Table 7 gives the unit costs of a hospital day, special services and diagnostic tests used for patients with iNTS. Table 8 gives the unit costs for the medication used. Table 9 gives the input data to estimate the ambulance service costs.

**Table 7: Data used to estimate the healthcare resource costs of the patients with iNTS in a specialised hospital.**

| **Healthcare resource** | **price** | | **unit** |
| --- | --- | --- | --- |
|  | **ETB** | **USD ^1^** |  |
| *Healthcare facility stay* |  |  |  |
| Costs per day ^1^ | 20 | 0.57 | /day |
| *Special services* |  |  |  |
| Special diet | 40 | 1.15 | /diet |
| Specialist consultation | 30 | 0.86 | /consult |
| Intravenous fluids | 50 | 1.43 | /bag |
| *Diagnostic tests* |  |  |  |
| Blood count: full count | 30 | 0.86 | /full count test |
| Blood count: Hb | 30 | 0.86 | /HB test |
| Blood count: HCT/PCV | 30 | 0.86 | /HCT/PCV test |
| Microbiology: gram stain/microscopy | 15 | 0.43 | / gram stain/microscopy test |
| Microbiology: culture | 30 | 0.86 | /culture test |
| Microbiology: sensitivity | 65 | 1.86 | /sensitivity test |
| Microbiology: antigen | 65 | 1.86 | /antigen test |
| Blood chemistry: electrolytes | 30 | 0.86 | /electrolyte test |
| Blood chemistry: glucose | 15 | 0.43 | /glucose test |

^1^ Using 2020 average official exchange rate of 34.93 ETB per USD (The World Bank, indicator code PA.NUS.FCRF). ^2^ Only out-of-pocket costs patients had to pay themselves for each day they stayed in the healthcare facility. These do not cover total costs for one hospital day.

**Table 8: Unit costs of medication used for patients with iNTS in a specialised hospital.**

| **Drug** | **route** | **dose amount** | **dose unit** | **Price per unit** | | **Unit** | **Unit amount** | **Dose unit** |
| --- | --- | --- | --- | --- | --- | --- | --- | --- |
|  |  |  |  | **ETB** | **USD^1^** |  |  |  |
| Azithromycin | Intravenous | 4 | g | 7 | 0.20 | /tab | 0.5 | g |
| Azithromycin | Injection | 200 | mg | 7 | 0.20 | /vial | 250 | mg |
| Ceftriaxone | Intravenous | 1 | g | 9 | 0.26 | /vial | 0.25 | g |
| Ciprofloxacin | Injection | 200 | mg | 5 | 0.14 | /vial | 200 | mg |
| Trimethoprim | Oral | 16 | mg | 4 | 0.11 | /tab | 100 | mg |

**Table 9: Data used to estimate ambulance service costs of patients with iNTS in a specialised hospital.**

|  | **price** | **unit** |
| --- | --- | --- |
| Ambulance service costs | 30 | ETB/km |
|  | 0.86 | USD/km ^1^ |
| Urban | 20 | km |
| Rural | 40 | km |

^1^ Using 2020 average official exchange rate of 34.93 ETB per USD (The World Bank, indicator code PA.NUS.FCRF).
