# Supplementary material for: Patient-related healthcare costs for diarrhoea, Guillain Barré syndrome and invasive non-typhoidal salmonellosis in Gondar, Ethiopia, 2020
Source: BMC Public Health. 2022 Nov 16;22:2091. doi: 10.1186/s12889-022-14539-1 (PMC9670532; doi:10.1186/s12889-022-14539-1)
Supplement: Supplementary file 3 — Additional file 3. Supplementary material S3. Healthcare resource use from patient records. [file 12889_2022_14539_MOESM3_ESM.docx]

**Supplementary material S3 to Direct and indirect (non)-medical patient-related healthcare costs for diarrhoea, GBS, and iNTS in three healthcare facilities in Gondar, Ethiopia, 2020**

**Healthcare resource use from patient records**

# Diarrhoea

Duration of hospital stay varied between one and ten days, with 75% staying for one day (Table 1). It should be noted that a duration of one day in this study includes admittance into the hospital from two hours up to 24 hours, which is recorded as hospital stay. Hospital stay fee is the same for all these patients. Only in the specialised hospital, seven patients stayed more than three days. Table 2 shows that patients’ average and range in length and weight per age was only recorded for children under 14 years.

**Table 1: Hospital stay duration of diarrhoea patients**

|  | **Health centre** | | **Private clinic** | | **Specialised hospital** | | **Total** | |
| --- | --- | --- | --- | --- | --- | --- | --- | --- |
| **Hospital stay (up to ... days)^1^** | **<5 years** | **≥5 years** | **<5 years** | **≥5 years** | **<5 years** | **≥5 years** | **<5 years** | **≥5 years** |
| 1 | 69 | 17 | 62 | 7 | 138 | 13 | 269 | 37 |
| 2 | 14 | 5 | 18 | 2 | 30 | 5 | 62 | 12 |
| 3 | 3 | 0 | 3 | 1 | 12 | 0 | 18 | 1 |
| 4 | 0 | 0 | 0 | 0 | 3 | 0 | 3 | 0 |
| 6 | 0 | 0 | 0 | 0 | 1 | 0 | 1 | 0 |
| 7 | 0 | 0 | 0 | 0 | 1 | 0 | 1 | 0 |
| 10 | 0 | 0 | 0 | 0 | 1 | 1 | 1 | 1 |

^1^ Note that a duration of one day includes stay from a few hours up to 24 hours. Hospital stay costs and related issues are similar for patients admitted from a few to 24 hours.

**Table 2: Summary of data on length and weight of diarrhoea patients per estimated age^1^**

|  | **Patients** | **Length (cm)** | | | **Weight (kg)** | | |
| --- | --- | --- | --- | --- | --- | --- | --- |
| **Age (years)** | **N** | **average** | **min** | **max** | **Average** | **min** | **max** |
| 0 | 32 | 67.1 | 47 | 82 | 7.5 | 4.5 | 12 |
| 1 | 73 | 69.7 | 38 | 90 | 8.3 | 4 | 15 |
| 2 | 47 | 80.7 | 58 | 167 | 9.5 | 4 | 14 |
| 3 | 20 | 87.1 | 50 | 102 | 11.4 | 3.5 | 15 |
| 4 | 8 | 108.6 | 67 | 200 | 13.9 | 5.5 | 21.5 |
| 5 | 7 | 103.9 | 92 | 113.5 | 13.7 | 7 | 18.1 |
| 6 | 1 | 82.0 | 82 | 82 | 10.0 | 10 | 10 |
| 8 | 6 | 121.3 | 115 | 127 | 20.1 | 18.5 | 22 |
| 9 | 1 | 110.0 | 110 | 110 | 16.5 | 16.5 | 16.5 |
| 10 | 3 | 131.3 | 121 | 148 | 25.7 | 20 | 32 |
| 13 | 1 | 134.0 | 134 | 134 | 24.0 | 24 | 24 |

^1^ For almost all patients, only year of birth was available in patient records. Assumed birth date was set at the first day of the year. Age (year) at admission was estimated as admission date – assumed birth date.

Before entering the healthcare facility, patients had on average 2.3 days of diarrhoea, with a median of 2 days and a range between 0 and 14 days (Table 3). Over 80% had between 0 and 3 days of diarrhoea before visiting the healthcare facility. Patients visiting the private clinic had less days of diarrhoea before visiting the healthcare facility (average 0.9, median 1 day) than the specialised hospital (average 2.7, median 2 days) and the health centre (average 2.8, median 3 days).

**Table 3: Patients with number of days with diarrhoea before entering the healthcare facility**

|  | **Number of patients** | | | |
| --- | --- | --- | --- | --- |
| **Number of preceding days with diarrhoea** | **Health centre** | **Private clinic** | **Specialised hospital** | **Total** |
| 0 | 0 | 18 | 4 | 22 |
| 1 | 28 | 68 | 71 | 167 |
| 2 | 24 | 5 | 37 | 66 |
| 3 | 34 | 2 | 44 | 80 |
| 4 | 7 | 0 | 19 | 26 |
| 5 | 4 | 0 | 13 | 17 |
| 6 | 2 | 0 | 6 | 8 |
| 7 | 8 | 0 | 5 | 13 |
| 8 | 1 | 0 | 2 | 3 |
| 10 | 0 | 0 | 2 | 2 |
| 12 | 0 | 0 | 1 | 1 |
| 14 | 0 | 0 | 1 | 1 |
| Average duration (days) | 2.8 | 0.9 | 2.7 | 2.3 |
| Median duration (days) | 3 | 1 | 2 | 2 |
| **Total** | **108** | **93** | **205** | **406** |

Less than 10% of the patients in the health centre and private clinic received care prior to visiting the healthcare facility, whereas this was over 20% in the specialised hospital (Table 4). No large differences were observed between male and female patients, nor between patients under 5 and of 5 years or older. For the specialised hospital, previously visited facilities included a primary hospital, health centre, or private health institution (Table 5). For the private clinic, this was a health centre. For the health centre, this were a traditional healer and a drug store.

**Table 4: Number of patients with and without care before they entered the healthcare facility**

|  | **Health centre** | | **Private clinic** | | **Specialised hospital** | | **Total** |
| --- | --- | --- | --- | --- | --- | --- | --- |
| **Prior care** | **<5 years** | **≥5 years** | **<5 years** | **≥5 years** | **<5 years** | **≥5 years** |  |
| No | 80 | 19 | 75 | 8 | 146 | 14 | 342 |
| Yes | 6 | 3 | 8 | 2 | 40 | 5 | 64 |
| **Total** | **86** | **22** | **83** | **10** | **186** | **19** | **406** |
|  |  |  |  |  |  |  |  |
| **Prior care** | **Female** | **Male** | **Female** | **Male** | **Female** | **Male** |  |
| No | 49 | 50 | 18 | 65 | 66 | 94 | 342 |
| Yes | 8 | 1 | 1 | 9 | 24 | 21 | 64 |
| **Total** | **57** | **51** | **19** | **74** | **90** | **115** | **406** |

**Table 5: Providers of care to the patients before the patients entered the hospital**

|  | **Number of patients** | | |
| --- | --- | --- | --- |
| **Care provider** | **Health centre** | **Private clinic** | **Specialised hospital** |
| Another similar health facility | 1 |  | 1 |
| Health centre | 1 | 5 | 14 |
| Herbalist |  | 1 |  |
| Over the counter drug | 3 |  |  |
| Primary hospital |  |  | 15 |
| Private health institution | 1 |  | 11 |
| Specialised hospital |  |  | 2 |
| Traditional healer | 3 | 3 |  |
| **Total** | **9** | **9** | **43** |

Admission diagnosis of almost 90% of patients was related to diarrhoea (Table 6). In the private clinic this was higher than in the health centre and specialised hospital. For 50% of the patients, final diagnosis was diarrhoea with some dehydration, 45% with no dehydration and 5% with severe dehydration (Table 7). In the health centre, almost twice as many patients had final diagnosis with no dehydration and half as many with some dehydration compared to the other two facilities. No clear pattern can be seen between admission and final diagnosis (Table 8). All patients left the healthcare facilities alive, with 75% well and 15% partially recovered (Table 9). Only nine people visiting the health centre had co-morbidities, and all had only one (Table 10).

**Table 6: Admission diagnosis patients with diarrhoea per healthcare facility**

| **Admission diagnosis** | **Health centre** | **Private clinic** | **Specialised hospital** | **Total** |
| --- | --- | --- | --- | --- |
| Diarrhoea | 19 | 88 | 126 | 233 |
| Non bloody diarrhoea | 64 | 4 | 18 | 86 |
| Bloody diarrhoea | 1 |  | 38 | 39 |
| Dysentery | 8 | 1 | 10 | 19 |
| Helminthiasis | 4 |  | 6 | 10 |
| Amebiasis | 6 |  | 2 | 8 |
| Giardia | 4 |  | 3 | 7 |
| Mucoid diarrhoea | 1 |  | 1 | 2 |
| Salmonellosis^1^ | 1 |  | 1 | 2 |
| **Total** | **108** | **93** | **205** | **406** |

^1^ It is uncommon that the admission diagnosis is salmonellosis.

**Table 7: Final diagnosis patients with diarrhoea per healthcare facility**

| **Final diagnosis** | **Health centre** | **Private clinic** | **Specialised hospital** | **Total** |
| --- | --- | --- | --- | --- |
| Diarrhoea with no dehydration | 70 | 27 | 79 | 176 |
| Diarrhoea with some dehydration | 33 | 57 | 120 | 210 |
| Diarrhoea with severe dehydration | 5 | 9 | 6 | 20 |
| **Grand Total** | **108** | **93** | **205** | **406** |

**Table 8: Admission and final diagnosis of the patients with diarrhoea over the three types of healthcare facilities**

|  | **Final diagnosis** | | |  |
| --- | --- | --- | --- | --- |
| **Admission diagnosis** | **Diarrhoea with no dehydration** | **Diarrhoea with some dehydration** | **Diarrhoea with severe dehydration** | **Total** |
| Amebiasis | 4 | 4 | 0 | 8 |
| Bloody diarrhoea | 11 | 25 | 3 | 39 |
| Diarrhoea | 95 | 129 | 9 | 233 |
| Dysentery | 6 | 9 | 4 | 19 |
| Giardia | 4 | 3 | 0 | 7 |
| Helminthiasis | 3 | 7 | 0 | 10 |
| Mucoid diarrhoea | 1 | 1 | 0 | 2 |
| Non bloody diarrhoea | 51 | 31 | 4 | 86 |
| Salmonellosis | 1 | 1 | 0 | 2 |
| **Total** | **176** | **210** | **20** | **406** |

**Table 9: Health outcome on discharge of the patients.**

| **Health outcome** | **Health centre** | **Private clinic** | **Specialised hospital** | **Total** |
| --- | --- | --- | --- | --- |
| Absconded | 0 | 0 | 1 | 1 |
| Alive and partially recovered | 15 | 12 | 36 | 63 |
| Alive and well | 82 | 65 | 151 | 298 |
| Alive, but outcome unknown/missing | 7 | 13 | 11 | 31 |
| Discharged with medical advice | 4 | 3 | 6 | 13 |
| **Total** | **108** | **93** | **205** | **406** |

**Table 10: Type and number of comorbidities of the patients.**

| **Co-morbidity** | **Health centre** | **Private clinic** | **Specialised hospital** | **Total** |
| --- | --- | --- | --- | --- |
| No co-morbidities | 99 | 93 | 205 | 397 |
| Acute respiratory infection | 1 | 0 | 0 | 1 |
| Dyspepsia | 1 | 0 | 0 | 1 |
| Giardia | 2 | 0 | 0 | 2 |
| Hypertension | 1 | 0 | 0 | 1 |
| Inflammatory bowel syndrome | 1 | 0 | 0 | 1 |
| Tonsillitis | 3 | 0 | 0 | 3 |
| **Total** | **108** | **93** | **205** | **406** |

The drugs used for diarrhoea patients were grouped in categories (Table 11). For detailed information about the drug use, see Table 12. Supportive drugs and antibiotics were used in many patients in all three healthcare facilities, but the exact type used differed. In the health centre, ORS was basically the only supportive drug used, whereas in the private clinic glucose 40% and metoclopramide and in the specialised hospital Ringer’s solution were used in addition to ORS. In the health centre the antibiotics cotrimoxazole and metronidazole were used for many patients, whereas in the private clinic these were ceftriaxone and ampicillin, and in the specialised hospital ceftriaxone and cotrimoxazole. Anti-inflammatory/pain relief drugs (Paracetamol) were used more in the health centre than in the other two facilities. Anti-inflammatory drugs were only used in the private clinic. Supportive drugs for the gastro-intestinal tract were used in the health centre and the private clinic. Anthelmintics were used in a few patients in the health centre and specialised hospital.

**Table 11: Number of patients that used drugs in each type of healthcare facility.**

| **Drug category** | **Drug** | **Health centre** | **Private clinic** | **Specialised hospital** | **Total** |
| --- | --- | --- | --- | --- | --- |
| Supportive | Glucose 40% | 1 | 41 |  | 42 |
|  | Ringer’s solution | 0 | 0 | 27 | 27 |
|  | Normal saline | 1 | 3 | 9 | 13 |
|  | ORS | 100 | 29 | 161 | 290 |
|  | Protec | 0 | 4 | 0 | 4 |
|  | Vitamin B complex | 1 | 0 | 0 | 1 |
|  | Zinc tablet | 63 | 13 | 138 | 214 |
| ***Supportive Total*** |  | ***166*** | ***90*** | ***335*** | ***591*** |
| Antibiotics | Amoxicillin | 3 | 1 | 7 | 11 |
|  | Ampicillin | 0 | 12 | 16 | 28 |
|  | Ceftriaxone | 1 | 81 | 45 | 127 |
|  | Ciprofloxacin | 10 | 0 | 7 | 17 |
|  | Cloxacillin | 0 | 3 | 1 | 4 |
|  | Cotrimoxazole | 60 | 1 | 25 | 86 |
|  | Gentamycin | 0 | 2 | 4 | 6 |
|  | Metronidazole | 20 | 9 | 5 | 34 |
| **Antibiotics Total** |  | **94** | **109** | **110** | **313** |
| Anti-inflammatory/pain relief | Paracetamol | 22 | 2 | 3 | 27 |
| ***Anti-inflammatory/pain relief Total*** |  | ***22*** | ***2*** | ***3*** | ***27*** |
| Supportive/Gastrointestinal tract | Metoclopramide | 3 | 37 | 0 | 40 |
|  | Omeprazole | 1 | 0 | 0 | 1 |
| ***Supportive/Gastrointestinal tract Total*** |  | ***4*** | ***37*** | ***0*** | ***41*** |
| Anthelmintics | Albendazole | 1 | 0 | 5 | 6 |
|  | Mebendazole | 6 | 1 | 3 | 10 |
|  | Praziquantel | 1 | 0 | 2 | 3 |
| ***Anthelmintics Total*** |  | ***8*** | ***1*** | ***10*** | ***19*** |
| Anti-inflammatory | Dexamethasone | 0 | 1 | 0 | 1 |
|  | Diclofenac | 0 | 7 | 0 | 7 |
| ***Anti-inflammatory Total*** |  | ***0*** | ***8*** | ***0*** | ***8*** |
| Antimalarial | Coartem | 1 | 0 | 0 | 1 |
| ***Antimalarial Total*** |  | ***1*** | ***0*** | ***0*** | ***1*** |

**Table 12: Number of patients with diarrhoea that used drugs in each type of healthcare facility including route, dose, unit, frequency and number of days of administering.**

|  |  |  |  |  |  | **Number of patients** | | |
| --- | --- | --- | --- | --- | --- | --- | --- | --- |
| **Drug** | **Route^a^** | **Dose** | **Unit** | **freq^b^** | **days^c^** | **Health centre** | **Private clinic** | **Specialised hospital** |
| Albendazole | O | 200 | mg | Once | 1 | 1 |  |  |
|  |  | 400 | mg | Once | 1 |  |  | 5 |
| Amoxicillin | O | 250 | mg | TID | 6 |  |  | 1 |
|  |  |  |  |  | 7 | 1 |  |  |
|  |  |  |  |  | 10 |  |  | 2 |
|  |  | 500 | mg | TID | 7 | 1 |  |  |
|  |  | 750 | mg | TID | 2 |  |  | 2 |
|  |  |  |  |  | 7 | 1 | 1 | 2 |
| Ampicillin | I | 100 | mg | QID | 3 |  |  | 2 |
|  |  | 137 | mg | QID | 1 |  |  | 1 |
|  |  | 150 | mg | QID | 1 |  | 2 |  |
|  |  | 188 | mg | QID | 3 |  |  | 1 |
|  |  | 200 | mg | QID | 1 |  | 4 |  |
|  |  |  |  |  | 6 |  |  | 1 |
|  |  | 225 | mg | QID | 5 |  |  | 1 |
|  |  | 250 | mg | QID | 1 |  | 1 |  |
|  |  |  |  |  | 3 |  |  | 2 |
|  |  |  |  |  | 5 |  |  | 1 |
|  |  |  |  |  | 6 |  |  | 1 |
|  |  | 300 | mg | QID | 1 |  | 1 |  |
|  |  | 325 | mg | QID | 1 |  |  | 1 |
|  |  |  |  |  | 5 |  |  | 1 |
|  |  | 350 | mg | QID | 1 |  | 1 |  |
|  |  |  |  |  | 3 |  |  | 2 |
|  |  | 400 | mg | QID | 3 |  |  | 1 |
|  |  | 425 | mg | QID | 5 |  |  | 1 |
|  |  | 500 | mg | QID | 1 |  | 3 |  |
| Coartem | O | 4 | mg | BID | 3 | 1 |  |  |
| Ceftriaxone | I | 100 | mg | BID | 3 |  |  | 1 |
|  |  | 112.5 | mg | BID | 3 |  |  | 1 |
|  |  | 125 | mg | BID | 2 |  |  | 4 |
|  |  |  |  |  | 3 |  | 8 |  |
|  |  |  |  |  | 5 |  | 1 |  |
|  |  |  |  |  | 7 |  | 1 |  |
|  |  | 137 | mg | BID | 3 |  |  | 1 |
|  |  | 138 | mg | BID | 3 |  |  | 1 |
|  |  | 150 | mg | BID | 3 |  | 4 | 2 |
|  |  | 160 | mg | BID | 2 |  |  | 1 |
|  |  | 162.5 | mg | BID | 3 |  |  | 1 |
|  |  | 175 | mg | BID | 2 |  |  | 1 |
|  |  |  |  |  | 3 |  |  | 1 |
|  |  | 200 | mg | BID | 2 |  |  | 2 |
|  |  |  |  |  | 3 |  | 2 | 1 |
|  |  | 210 | mg | BID | 3 |  |  | 1 |
|  |  | 213 | mg | BID | 3 |  |  | 1 |
|  |  | 250 | mg | Daily | 3 |  |  | 1 |
|  |  |  |  | BID | 2 |  |  | 2 |
|  |  |  |  |  | 3 | 1 | 6 | 6 |
|  |  | 262 | mg | BID | 2 |  |  | 1 |
|  |  | 275 | mg | BID | 2 |  |  | 1 |
|  |  |  |  |  | 3 |  |  | 1 |
|  |  | 300 | mg | BID | 2 |  |  | 1 |
|  |  |  |  |  | 3 |  | 13 |  |
|  |  | 325 | mg | BID | 3 |  |  | 2 |
|  |  | 350 | mg | BID | 2 |  | 1 |  |
|  |  |  |  |  | 3 |  | 14 | 1 |
|  |  | 400 | mg | BID | 1 |  | 3 |  |
|  |  |  |  |  | 3 |  | 17 | 1 |
|  |  |  |  |  | 5 |  | 1 |  |
|  |  | 450 | mg | BID | 2 |  |  | 1 |
|  |  |  |  |  | 3 |  |  | 1 |
|  |  | 475 | mg | BID | 3 |  |  | 1 |
|  |  | 500 | mg | BID | 3 |  | 9 | 2 |
|  |  | 550 | mg | BID | 2 |  |  | 1 |
|  |  | 750 | mg | BID | 3 |  | 1 | 3 |
| Ciprofloxacin | O | 250 | mg | BID | 3 | 8 |  | 6 |
|  |  |  |  |  | 7 | 2 |  | 1 |
| Cloxacillin | I | 200 | mg | Daily | 1 |  | 1 |  |
|  |  | 250 | mg | BID | 1 |  | 1 |  |
|  |  | 300 | mg | Daily | 1 |  | 1 |  |
|  |  | 500 | mg | BID | 1 |  |  | 1 |
| Cotrimoxazole | O | 200 | mg | BID | 7 | 2 | 1 |  |
|  |  | 220 | mg | BID | 5 | 1 |  |  |
|  |  | 240 | mg | BID | 2 | 3 |  |  |
|  |  |  |  |  | 5 | 2 |  |  |
|  |  |  |  |  | 6 | 1 |  |  |
|  |  |  |  |  | 7 | 45 |  | 18 |
|  |  |  |  |  | 8 | 2 |  |  |
|  |  | 250 | mg | BID | 7 | 2 |  | 1 |
|  |  | 280 | mg | BID | 7 |  |  | 1 |
|  |  | 300 | mg | BID | 7 |  |  | 1 |
|  |  | 350 | mg | BID | 7 |  |  | 1 |
|  |  |  |  |  | 10 |  |  | 1 |
|  |  | 375 | mg | BID | 7 |  |  | 1 |
|  |  | 480 | mg | BID | 5 | 1 |  |  |
|  |  |  |  |  | 7 |  |  | 1 |
|  |  | 960 | mg | BID | 7 | 1 |  |  |
| Dexamethasone | I | 4 | mg | BID | 1 |  | 1 |  |
| Diclofenac | Inj | 2 | mg | Once | 1 |  | 3 |  |
|  |  | 2.5 | mg | Once | 1 |  | 1 |  |
|  |  | 5 | mg | Once | 1 |  | 3 |  |
| Gentamycin | I | 10 | mg | BID | 1 |  | 1 |  |
|  |  | 40 | mg | BID | 3 |  |  | 1 |
|  |  | 150 | mg | BID | 3 |  | 1 |  |
|  |  | 230 | mg | BID | 5 |  |  | 1 |
|  |  | 250 | mg | BID | 5 |  |  | 1 |
|  |  | 325 | mg | BID | 5 |  |  | 1 |
| Glucose 40% | I | 2 | l | AN | 1 | 1 | 11 |  |
|  |  |  | ml | AN | 1 |  | 20 |  |
|  |  |  |  | Once | 1 |  | 9 |  |
|  |  | 3 | l | AN | 1 |  | 1 |  |
| Ringer’s solution | I | 450 | ml | Once | 1 |  |  | 1 |
|  |  | 490 | ml | Once | 1 |  |  | 1 |
|  |  | 500 | ml | Once | 1 |  |  | 2 |
|  |  | 525 | ml | Once | 1 |  |  | 4 |
|  |  | 560 | ml | Once | 1 |  |  | 1 |
|  |  | 562 | ml | Once | 1 |  |  | 1 |
|  |  | 600 | ml | Once | 1 |  |  | 1 |
|  |  | 650 | ml | Once | 1 |  |  | 1 |
|  |  | 712.5 | ml | Once | 1 |  |  | 1 |
|  |  | 750 | ml | Once | 1 |  |  | 10 |
|  |  | 900 | ml | Once | 1 |  |  | 2 |
|  |  | 1200 | ml | Once | 1 |  |  | 1 |
|  |  | 1500 | ml | Once | 1 |  |  | 1 |
| Mebendazole | O | 100 | mg | BID | 3 | 3 |  |  |
|  |  |  |  |  | 4 | 1 |  |  |
|  |  |  |  |  | 7 | 1 |  |  |
|  |  | 200 | mg | BID | 3 | 1 | 1 | 3 |
| Metoclopramide | O | 0.75 | mg | BID | 1 |  | 1 |  |
|  |  | 1 | mg | Once | 1 |  | 1 |  |
|  |  | 1.2 | mg | Once | 1 |  | 1 |  |
|  |  | 1.5 | mg | Once | 1 |  | 1 |  |
|  |  | 2 | mg | Once | 1 |  | 9 |  |
|  |  | 2.5 | mg | Once | 1 |  | 4 |  |
|  |  | 5 | mg | Once | 1 |  | 18 |  |
|  |  |  |  | BID | 1 | 1 |  |  |
|  |  |  |  |  | 5 | 1 |  |  |
|  |  |  |  |  | 6 | 1 |  |  |
|  |  | 120 | ml | Once | 1 |  | 2 |  |
| Metronidazole | O | 100 | mg | BID | 7 |  | 1 |  |
|  |  | 125 | mg | TID | 7 | 2 |  | 1 |
|  |  |  |  | BID | 7 | 3 | 3 |  |
|  |  | 150 | mg | BID | 7 |  | 3 |  |
|  |  | 200 | mg | TID | 7 |  | 1 |  |
|  |  | 250 | mg | TID | 7 | 1 |  |  |
|  |  |  |  | BID | 7 | 5 |  | 2 |
|  |  | 300 | mg | BID | 7 |  |  | 1 |
|  |  | 350 | mg | BID | 7 |  | 1 | 1 |
|  |  | 500 | mg | TID | 7 | 8 |  |  |
|  |  | 750 | mg | TID | 7 | 1 |  |  |
| Normal saline | I | 150 | ml | AN | 1 |  | 1 |  |
|  |  | 200 | ml | AN | 2 |  |  | 1 |
|  |  | 250 | ml | AN | 2 |  |  | 3 |
|  |  | 300 | ml | AN | 2 |  |  | 4 |
|  |  | 500 | ml | AN | 1 |  | 1 |  |
|  |  | 550 | ml | AN | 2 |  |  | 1 |
|  |  | 600 | ml | AN | 1 |  | 1 |  |
|  |  | 1000 | ml | AN | 1 | 1 |  |  |
| Omeprazole | O | 20 | mg | TID | 10 | 1 |  |  |
| ORS | N | 100 | ml | AN | 1 |  |  | 1 |
|  |  | 450 | ml | AN | 1 |  |  | 2 |
|  |  | 525 | ml | AN | 1 |  |  | 1 |
|  |  | 600 | ml | AN | 1 |  |  | 1 |
|  |  | 675 | ml | AN | 1 |  |  | 1 |
|  |  | 750 | ml | AN | 1 |  |  | 10 |
|  |  | 1000 | ml | AN | 1 |  |  | 1 |
|  | O | 1 | l | AN | 1 |  |  | 55 |
|  |  |  |  |  | 2 |  | 11 | 8 |
|  |  | 2 | l | AN | 1 | 5 | 5 | 7 |
|  |  |  |  |  | 2 | 92 | 13 | 57 |
|  |  |  |  |  | 3 | 3 |  |  |
|  |  |  |  | BID | 2 |  |  | 1 |
|  |  | 3 | l | AN | 1 |  |  | 1 |
|  |  |  |  |  | 2 |  |  | 14 |
|  |  |  |  | BID | 2 |  |  | 1 |
| Paracetamol | O | 120 | mg | AN | 1 | 16 | 2 | 2 |
|  |  |  |  |  | 2 | 1 |  |  |
|  |  | 500 | mg | AN | 1 | 4 |  |  |
|  |  |  |  |  | 2 | 1 |  |  |
|  | R | 82 | mg | AN | 1 |  |  | 1 |
| Praziquantel | O | 200 | mg | Once | 1 | 1 |  |  |
|  |  | 600 | mg | Once | 1 |  |  | 2 |
| Protec | Inj | 400 | mg | BID | 1 |  | 2 |  |
|  | O | 500 | mg | BID | 1 |  | 2 |  |
| Vitamin B complex | I | 2 | ampule | Daily | 1 | 1 |  |  |
| Zinc tablet | O | 10 | mg | AN | 10 |  |  | 2 |
|  |  |  |  | Daily | 2 | 1 |  |  |
|  |  |  |  |  | 5 |  | 1 | 1 |
|  |  |  |  |  | 6 |  |  | 1 |
|  |  |  |  |  | 7 |  |  | 9 |
|  |  |  |  |  | 10 | 7 |  | 3 |
|  |  |  |  |  | 14 |  |  | 1 |
|  |  | 20 | mg | AN | 10 |  |  | 4 |
|  |  |  |  | Daily | 2 | 14 |  |  |
|  |  |  |  |  | 5 |  | 2 |  |
|  |  |  |  |  | 7 |  |  | 4 |
|  |  |  |  |  | 10 | 41 | 10 | 111 |
|  |  |  |  |  | 14 |  |  | 1 |
|  |  | 75 | mg | Daily | 7 |  |  | 1 |

^a^ O = Oral, I = Intravenous, Inj = Injection, N = N, R = R. ^b^ Frequency: Once = Taken once, D = daily, BID = Two times a day, TID = Three times a day, QID = Four times a day, AN = As needed. ^c^ Number of days the drug was prescribed including days during hospitalization and days after discharge.

At least one stool test was used for more than 90% of the patients in all three healthcare facilities (Table 13). At least one blood test was used for 70% of the patients in the private and specialised hospital, whereas in the health centre it was hardly used. A urinalysis was used for about 50% of the patients in the specialised hospital, but for less than 5% in the private clinic and the health centre. Blood culture was only used for 5% or less of the patients in all three facilities.

**Table 13: Number of patients that used diagnostic tests.**

|  | **number of times a test was performed** | | | | | |
| --- | --- | --- | --- | --- | --- | --- |
|  | **0** | **1** | **2** | **3** | **4** | **5** |
| *Health Centre* |  |  |  |  |  |  |
| Blood test | 104 | 1 | 0 | 0 | 0 | 0 |
| Blood culture | 104 | 1^1^ | 0 | 0 | 0 | 0 |
| Urinalysis | 102 | 3 | 0 | 0 | 0 | 0 |
| Stool test | 1 | 90 | 14 | 0 | 0 | 0 |
| *Private clinic* |  |  |  |  |  |  |
| Blood test | 27 | 64 | 2 | 0 | 0 | 0 |
| Blood culture | 93 | 0 | 0 | 0 | 0 | 0 |
| Urinalysis | 89 | 4 | 0 | 0 | 0 | 0 |
| Stool test | 4 | 86 | 3 | 0 | 0 | 0 |
| *Specialised hospital* |  |  |  |  |  |  |
| Blood test | 60 | 79 | 41 | 13 | 8 | 4 |
| Blood culture | 194 | 11 | 0 | 0 | 0 | 0 |
| Urinalysis | 102 | 99 | 3 | 1 | 0 | 0 |
| Stool test | 18 | 177 | 8 | 0 | 0 | 1 |

^1^ It is uncommon that a blood culture test can be done in a health centre.

We collected data on the potential etiology for diarrhoea from patient records. Non-typhoidal *Salmonella enterica* was indicated as the cause for diarrhoea in about 35% of the patients, ETEC in about 22%, and *Campylobacter* spp. in about 14% (Table 14). In the private clinic, for relatively many patients the cause was non-typhoidal *Salmonella enterica* compared to the health centre and specialised hospital. These last two healthcare facilities had relatively many patients with *Entamoeba histolytica* compared to the private clinic. They also had more patients with no information about the etiology. For example, many red blood cells and pus cells were recorded as cause. However, many red blood cells and pus cells is unlikely to be the cause for diarrhoea, because it is more a result of an infection than a cause for it. It was not possible to accurately link many red blood and pus cells to a specific cause. That many red blood cells and pus cells was found in patient records could be due to inaccurate data recording. Such inaccuracy in data recording might result from physicians taking more care and attention in clearly writing their prescriptions than in writing test results on lab result sheets. If such a lab result sheet is unreadable or even lost, nurses or healthcare workers transferring the results to the patient’s record could record whatever they feel appropriate, such as many red blood cells or pus cells.

**Table 14: Etiology of diarrhoea in each type of healthcare facility as recorded in patient records.**

| **Etiology** | **Health centre** | | **Private clinic** | | **Specialised hospital** | | **Total** | |
| --- | --- | --- | --- | --- | --- | --- | --- | --- |
|  | **N** | **%** | **N** | **%** | **N** | **%** | **N** | **%** |
| Non-typhoidal *Salmonella enterica* ^1^ | 25 ^2^ | 23 | 50 | 54 | 67 | 33 | 142 | 35 |
| Enterotoxigenic *Escherichia coli* | 22 ^2^ | 20 | 24 | 26 | 45 | 22 | 91 | 22 |
| *Campylobacter* spp. | 10 ^2^ | 9 | 17 | 18 | 29 | 14 | 56 | 14 |
| *Entamoeba histolytica* | 13 | 12 | 0 | 0 | 6 | 3 | 19 | 5 |
| *Giardia lamblia* | 7 | 6 | 0 | 0 | 0 | 0 | 7 | 2 |
| Many red blood cells and pus cells | 13 | 12 | 0 | 0 | 46 | 22 | 59 | 15 |
| Missing | 18 | 17 | 2 | 2 | 12 | 6 | 32 | 8 |
| **Total** | **108** |  | **93** |  | **205** |  | **406** |  |

^1^ The etiology recorded was non-typhoidal *Salmonella enterica* for all patients except for one patient at the health centre for which salmonella was recorded. ^b^ It is uncommon that tests for these pathogens can be done in a health centre.

In all three healthcare facilities, most patients stayed in the pediatric ward or outpatient clinic (Table 15). Most patients stayed one day only. Average total stay was lowest with 1.2 days in the health centre and with 1.5 days highest in the specialised hospital.

**Table 15: Duration of stay in different wards of the patients (in days).**

|  | **Health centre** | | | **Private clinic** | | | **Specialised hospital** | | |
| --- | --- | --- | --- | --- | --- | --- | --- | --- | --- |
|  |  | **Days** | |  | **Days** | |  | **Days** | |
|  | **N** | **average (st.dev)** | **Min – max** | **N** | **average (st.dev)** | **Min – max** | **N** | **average (st.dev)** | **Min – max** |
| Outpatient clinic | 21 | 1.0 (0.0) | 1 - 1 | 32 | 1.0 (0.0) | 1 - 1 | 62 | 1.0 (0.0) | 1 - 1 |
| Pediatric ward | 88 | 1.1 (0.3) | 1 - 3 | 74 | 1.0 (0.2) | 1 - 2 | 68 | 1.2 (1.1) | 1 - 10 |
| Intensive care unit / special care baby unit | 0 |  |  | 0 |  |  | 1 | 2.0 (N.a. ^1^) | 2 - 2 |
| Emergency room | 11 | 1.2 (0.4) | 1 - 2 | 12 | 1.1 (0.3) | 1 - 2 | 114 | 1.4 (1.2) | 1 - 10 |
| Antenatal care | 4 | 1.0 (N.a.) | 1 - 1 | 0 |  |  | 0 |  |  |
| Total stay | 108 | 1.2 (0.5) | 1 - 3 | 93 | 1.3 (0.5) | 1 - 3 | 205 | 1.5 (1.2) | 1 - 10 |

^1^ Not available.

Table 16 shows that intravenous fluids were used most for most patients in all three types of hospital. Special diet was only used for patients in the health centre and specialised hospital. The Cardiopulmonary resuscitation and autopsy were not used.

**Table 16: Number of diarrhoea patients that used other services.**

|  | **number of times a service was used** | | | | |
| --- | --- | --- | --- | --- | --- |
|  | **0** | **1** | **2** | **3** | **4** |
| *Health Centre* |  |  |  |  |  |
| Special diet | 82 | 25 | 1 |  |  |
| Specialist consultation | 85 | 22 | 1 |  |  |
| Intravenous fluids | 24 | 50 | 34 |  |  |
| *Private clinic* |  |  |  |  |  |
| Special diet | 93 |  |  |  |  |
| Specialist consultation | 82 | 11 |  |  |  |
| Intravenous fluids | 24 | 26 | 30 | 12 | 1 |
| *Specialised hospital* |  |  |  |  |  |
| Special diet | 173 | 31 | 1 |  |  |
| Specialist consultation | 149 | 54 | 2 |  |  |
| Intravenous fluids | 25 | 109 | 66 | 4 | 1 |

Table 17 shows that about 30% of the patients in our sample used ambulance service. About two third of the patients in the health centre used ambulance services and about a quarter of the patients in the specialised hospital. The private clinic did not have an ambulance service available.

**Table 17: Number of diarrhoea patients that used ambulance service per healthcare facility.**

|  | **Number of times a service was used** | | | **Total** |
| --- | --- | --- | --- | --- |
|  | **0** | **1** | **2** |  |
| Health centre | 42 | 65 | 1 | 108 |
| Private clinic | 93 |  |  | 93 |
| Specialised hospital | 152 | 51 | 2 | 205 |
| Total | 287 | 116 | 3 | 406 |

Average costs of bringing a patient to the hospital was 63 ETB for one way (Table 18). Bus/train and car trips were most expensive, since these were used for longer travelling times of one hour and more. Taxi and foot trips were used for shorter travelling times. Half of the people used a taxi to bring a patient to the hospital. Average travelling time to bring a patient to the hospital (single trip) was 49 minutes (standard deviation 47, range 10 to 210). A similar transport means was used to bring the patient to the hospital and to visit the patient. The travelling costs of a person for visiting a patient were double that of the single way trip to bring the patient to the hospital. For visiting a patient, between 1 and 4 person roundtrips were made (Table 19). The total number of person roundtrips for bringing and visiting the patient were between 2 and 5 per patient. The correlation between number of person roundtrips for visiting a patient and duration of stay in the hospital was 0.15.

**Table 18: Transport means and costs to bring the patient to the hospital and to visit the patient**

|  | **Bring patient (one way)** | | **Visit patient (round trip)** | |
| --- | --- | --- | --- | --- |
| **Transport means** | **Respondents (N)** | **Average costs (ETB)** | **Respondents (N)** | **Average costs (ETB)** |
| Taxi | 12 | 23 | 13 | 46 |
| Bus/train | 5 | 184 | 4 | 420 ^1^ |
| Car | 2 | 47 | 3 | 116 |
| By foot | 1 | 0 | 1 | 0 |
| Bajaj contract | 1 | 30 | 0 | 0 |
| **Total** | **21** | **63** | **21** | **125** |

^1^ This number if high, because one patient had costs of 540 ETB per roundtrip. All the other patients going by bus/train had costs of 80 to 120 ETB per roundtrip.

**Table 19: Person roundtrips to visit patient**

|  | **Number of person roundtrips** | | | | |
| --- | --- | --- | --- | --- | --- |
|  | **1** | **2** | **3** | **4** | **Total** |
| Number of responses | 2 | 9 | 7 | 3 | 21 |

# Guillain-Barré syndrome

At admission, of the patients with GBS, five were between 20 and 30 years of age, twelve between 30 and 60 years, and four between 60 and 70 years. Patients stayed on average 21 days in the hospital (range 3 to 38) (Table 20). On average, patients were ill for 12 days before entering the hospital (0 to 30 days, standard deviation 8.8). We did not ask for the type of illness. 17 patients received care before entering the hospital, all from only one provider (Table 21). The most used providers were a health centre, a primary hospital and a private health institution.

**Table 20: Hospital stay duration of GBS patients**

| Duration hospital stay (days) | Mean 20.8 | Standard deviation 9.0 | Range 3-37 |
| --- | --- | --- | --- |

**Table 21: Providers of care to the patients before the patients entered the hospital**

| **Care provider** | **Number of patients** |
| --- | --- |
| Traditional healer | 2 |
| Herbalist | 1 |
| Health facility/health post | 2 |
| Health centre | 4 |
| Primary hospital | 4 |
| Private health institution | 4 |
| **Total** | **17** |

Admission diagnosis of 18 patients was related to a form of paralysis and of two to GBS (Table 22). Twelve patients had the final diagnosis mild GBS and nine severe GBS. At discharge, nine patients were fully recovered, six partially recovered, two had died and the rest was discharged with medical advice or referred to another hospital (Table 23). Ten patients had comorbidities, of which three two or three (Table 24). The most common comorbidities were related to the respiratory tract.

**Table 22: Admission and final diagnosis of the GBS patients**

|  | **Final diagnosis** | |  |
| --- | --- | --- | --- |
| **Admission diagnosis** | **Mild GBS** | **Severe GBS** | **Total** |
| Paraparesis | 3 | 1 | 4 |
| Nephrotic syndrome | 1 | 0 | 1 |
| Quadriplegia | 3 | 5 | 8 |
| Flaccid paraplegia | 1 | 0 | 1 |
| Flaccid quadriplegia and aspiration pneumonia | 0 | 1 | 1 |
| Hemiparesis | 3 | 1 | 4 |
| GBS | 1 | 1 | 2 |
| **Total** | **12** | **9** | **21** |

**Table 23: Health outcome on discharge of the GBS patients.**

| **Health outcome on discharge** | **Number of patients** |
| --- | --- |
| Alive and well | 9 |
| Alive and partially recovered | 6 |
| Died | 2 |
| Referred | 1 |
| Discharged with medical advice | 3 |
| **Total** | **21** |

**Table 24: Type and number of comorbidities of the GBS patients.**

| **Comorbidity** | **Number of patients** |
| --- | --- |
| No comorbidities | 11 |
| Nephrotic syndrome | 1 |
| Hypertension | 1 |
| Community acquired pneumonia | 2 |
| Urinary tract infection | 1 |
| Deep vein thrombosis | 1 |
| Hospital acquired pneumonia | 1 |
| Cardiac problem, Globoid Cell Leukodystrophy | 1 |
| Deep vein thrombosis, Healthcare associated pneumonia | 1 |
| Aspiration pneumonia, acute respiratory distress syndrome, hypothermia | 1 |

GBS patients stayed on average 22.1 days in the hospital (standard deviation 9.3 days) (Table 25). They stayed 18.2 days (standard deviation 8.1, range 2 to 32 days) in an intensive care unit and 3.9 days (standard deviation 2, range 0 to 9 days) in an emergency room. All patients used a specialist consultation, ranging from 1 to 8 times (Table 26). Most patients used had a special diet and used intravenous fluids, mostly only once. Cardiopulmonary resuscitation and autopsy were hardly used.

**Table 25: Duration of stay in different wards of the GBS patients (in days).**

| **Ward/unit ^1^** | **Average** | **Standard deviation** | **Minimum** | **Maximum** |
| --- | --- | --- | --- | --- |
| Emergency room | 3.9 | 2.1 | 0 | 9 |
| Intensive care unit | 18.2 | 8.1 | 2 | 32 |
| Total stay | 22.1 | 9.3 | 3 | 37 |

^1^ No stays at outpatient ward, pediatric ward, isolation unit or other locations.

**Table 26: Number of GBS patients that used special services.**

|  | **Number of times used** | | | | | | |
| --- | --- | --- | --- | --- | --- | --- | --- |
| **Special service** | **Did not use** | **1** | **2** | **3** | **4** | **8** | **10** |
| Special diet | 13 | 8 |  |  |  |  |  |
| Intravenous fluids | 1 | 17 | 2 |  |  |  | 1 |
| Cardiopulmonary resuscitation | 20 |  |  | 1 |  |  |  |
| Autopsy | 21 |  |  |  |  |  |  |

All patients used at least one diagnostic test (Table 27). Test most used were full blood count, blood electrolytes, blood glucose, and X-ray. Most diagnostic tests were only applied once for a patient. Both IVIg and methyl prednisolone treatment were given to two patients each (Table 28).

**Table 27: Number of GBS patients that used diagnostic tests.**

|  | **Number of times performed** | | | | |
| --- | --- | --- | --- | --- | --- |
| **Diagnostic test** | **Did not use** | **1** | **2** | **3** | **4** |
| Blood count |  |  |  |  |  |
| - full count |  | 21 |  |  |  |
| - Hb | 8 | 13 |  |  |  |
| - HCT/PCV | 7 | 14 |  |  |  |
| Blood culture | 12 | 9 |  |  |  |
| HIV test |  |  |  |  |  |
| - rapid test | 7 | 14 |  |  |  |
| - Elisa | 21 |  |  |  |  |
| Other microbiological tests |  |  |  |  |  |
| - gram stain/Microscopy | 11 | 9 | 1 |  |  |
| - culture | 10 | 11 |  |  |  |
| - sensitivity | 18 | 3 |  |  |  |
| - antigen | 12 | 9 |  |  |  |
| Radiology test: |  |  |  |  |  |
| - CT scan | 15 | 6 |  |  |  |
| - Ultrasound | 7 | 13 | 1 |  |  |
| - Other X-ray | 2 | 17 |  | 1 | 1 |
| Blood chemistry test |  |  |  |  |  |
| - Electrolytes |  | 21 |  |  |  |
| - Glucose | 1 | 20 |  |  |  |
| Spinal puncture test | 21 |  |  |  |  |
| EMG test | 21 |  |  |  |  |
| MRI | 15 | 6 |  |  |  |
| ECG | 17 | 4 |  |  |  |

**Table 28: Number of GBS patients that used further treatments.**

|  | **Number of times performed** | | |
| --- | --- | --- | --- |
| **Treatment** | **Did not use** | **1** |  |
| IVIg | 19 | 2 |  |
| Methyl prednisolone | 19 | 2 |  |

All patients consulted a neurologist, between 1 and 10 times (Table 29). Twenty patients consulted a physiotherapist, between 2 and 28 times. Nineteen patients consulted a nurse, between 3 and 30 times.

**Table 29: Number of GBS patients that used specialist consultation.**

|  | **Number of times used** | | | | | | | | | | | | | | | | | |
| --- | --- | --- | --- | --- | --- | --- | --- | --- | --- | --- | --- | --- | --- | --- | --- | --- | --- | --- |
| **Specialist** | **No use** | **1** | **2** | **3** | **4** | **5** | **6** | **7** | **8** | **9** | **10** | **14** | **15** | **18** | **20** | **25** | **28** | **30** |
| Physiotherapist | 1 |  | 1 | 1 | 2 | 1 | 1 | 1 | 1 |  | 3 | 1 | 3 | 1 | 3 |  | 1 |  |
| Neurologist |  | 1 | 4 | 2 | 3 | 1 | 4 |  | 2 | 1 | 3 |  |  |  |  |  |  |  |
| Rehabilitation | 21 |  |  |  |  |  |  |  |  |  |  |  |  |  |  |  |  |  |
| Nurse | 2 |  |  | 1 |  |  | 2 |  |  |  | 5 |  | 2 |  | 4 | 3 |  | 2 |

Drugs used for patients with GBS were categorized in antibiotics, anti-inflammatory, supportive, Supportive/Gastrointestinal tract, Anti-inflammatory/antidepressant, and pain relief (Table 30). Detailed information on routes, doses, frequency, and number of days of administering for each drug can be found in Table 31. All patients received antibiotics and most anti-inflammatory drugs as well. Different antibiotics and anti-inflammatory drugs were administered, with half the patients receiving ceftriaxone and dexamethasone. About half the patients received supportive drugs, mainly UFH, and supportive drugs for the gastrointestinal tract. Some patients also received anti-inflammatory/antidepressant and pain relief drugs.

**Table 30: Number of GBS patients that used drugs.**

| **Drug category** | **Drug** | **Number of patients** |
| --- | --- | --- |
| Antibiotics | Azithromycin | 1 |
|  | Cefepime | 1 |
|  | Ceftazidime | 1 |
|  | Ceftriaxone | 10 |
|  | Metronidazole | 4 |
|  | Vancomycin | 4 |
| ***Antibiotics Total*** |  | ***21*** |
| Anti-inflammatory | Acyclovir | 4 |
|  | Dexamethasone | 11 |
|  | Diclofenac | 1 |
|  | Prednisolone | 1 |
| ***Anti-inflammatory Total*** |  | ***17*** |
| Supportive | Enalapril | 2 |
|  | Lasix | 1 |
|  | Potassium chloride | 1 |
|  | UFH | 6 |
| ***Supportive Total*** |  | ***10*** |
| Supportive/Gastrointestinal tract | Bisacodyl | 3 |
|  | Lactulose | 1 |
|  | Omeprazole | 4 |
| ***Supportive/Gastrointestinal tract Total*** |  | ***8*** |
| Anti-inflammatory/antidepressant | Amitriptyline | 6 |
| ***Anti-inflammatory/antidepressant Total*** |  | ***6*** |
| Pain relief | Tramadol | 3 |
| ***Pain relief Total*** |  | ***3*** |

**Table 31: Detailed information about drug used, route, dose, frequency, and number of days of administering for patients with GBS.**

|  |  |  |  |  | **Number of days** | | | | | | | | | | | |
| --- | --- | --- | --- | --- | --- | --- | --- | --- | --- | --- | --- | --- | --- | --- | --- | --- |
| **Drug** | **Route^a^** | **Dose** | **Unit** | **Freq.** | **2** | **3** | **4** | **5** | **6** | **7** | **10** | **12** | **14** | **16** | **21** | **30** |
| Acyclovir | I | 500 | mg | TID |  |  |  |  |  |  |  |  |  | 1 | 1 |  |
|  | O | 300 | mg | TID |  |  |  |  |  |  |  |  | 1 |  |  |  |
|  | O | 500 | mg | TID |  |  |  |  |  |  | 1 |  |  |  |  |  |
| Amitriptyline | O | 12.5 | mg | BID |  | 1 |  |  |  |  |  |  | 2 |  |  |  |
|  | O |  |  | Daily |  |  |  |  |  | 1 | 1 |  |  |  |  | 1 |
| Azithromycin | O | 500 | mg | Daily |  | 1 |  |  |  |  |  |  |  |  |  |  |
| Bisacodyl | O | 5 | mg | Daily | 2 |  |  | 1 |  |  |  |  |  |  |  |  |
| Cefepime | I | 2 | g | BID |  |  |  |  |  |  |  |  | 1 |  |  |  |
| Ceftazidime | I | 2 | g | TID |  |  |  |  |  |  |  |  | 1 |  |  |  |
| Ceftriaxone | I | 1 | g | BID |  |  |  |  | 1 | 2 | 1 | 1 | 3 |  |  |  |
|  | I | 2 | g | BID |  |  |  |  |  |  | 1 |  | 1 |  |  |  |
| Dexamethasone | I | 4 | mg | QID |  |  |  |  |  | 1 |  | 1 |  |  |  |  |
|  | I |  |  | TID |  |  |  |  |  |  |  |  | 2 |  |  |  |
|  | I | 8 | mg | QID |  |  |  |  |  |  |  | 1 |  | 1 | 1 |  |
|  | I |  |  | TID |  |  |  |  |  |  | 1 |  | 1 |  | 1 |  |
|  | I | 12 | mg | QID |  |  |  |  |  |  |  |  |  |  |  | 1 |
| Diclofenac | O | 50 | mg | BID |  |  |  |  |  | 1 |  |  |  |  |  |  |
| Enalapril | O | 2.5 | mg | BID |  |  |  |  |  |  |  |  | 1 |  |  |  |
|  | O |  |  | Daily |  |  |  |  | 1 |  |  |  |  |  |  |  |
| Lactulose | O | 45 | ml | Daily |  |  | 1 |  |  |  |  |  |  |  |  |  |
| Lasix | I | 80 | mg | TID |  |  |  |  |  |  |  |  | 1 |  |  |  |
| Metronidazole | I | 500 | mg | TID |  |  |  |  |  | 2 |  |  | 1 |  |  |  |
|  | O | 500 | mg | TID |  |  |  |  |  |  |  |  | 1 |  |  |  |
| Omeprazole | O | 20 | mg | BID | 2 |  |  |  |  |  | 1 |  | 1 |  |  |  |
| Potassium chloride | I | 40 | mmol | TID |  | 1 |  |  |  |  |  |  |  |  |  |  |
| Prednisolone | O | 50 | mg | Daily |  |  |  |  |  |  |  |  | 1 |  |  |  |
| Tramadol | I | 50 | mg | TID |  |  |  |  |  |  | 3 |  |  |  |  |  |
| UFH | S | 5000 | IU | BID | 1 |  |  | 1 |  | 1 | 3 |  |  |  |  |  |
| Vancomycin | I | 1 | g | BID |  |  |  |  |  |  |  |  | 2 |  |  |  |
|  |  | 250 | mg | BID |  |  |  |  |  |  | 1 |  | 1 |  |  |  |

^a^ I=intravenous, O=oral, S=subcutaneous.

Three of the patients experienced complications during the stay in the hospital, one cardiac arrest, one ARDS, and one deep venous thrombosis (Table 32). Table 33 shows that most of the 21 GBS patients used ambulance services, mostly only once about.

**Table 32: Patients with complications.**

| **Complication** | **Number of patients** |
| --- | --- |
| Cardiac arrest | 1 |
| ARDS | 1 |
| Deep venous thrombosis | 1 |
| No complications | 18 |

**Table 33: Number of GBS patients that used other services.**

|  | **Did not use** | **Used 1 time** | **Used 2 times** |
| --- | --- | --- | --- |
| Ambulance service | 2 | 18 | 1 |

# Invasive non-typhoidal salmonellosis

Four patients were younger than 5 years, nine were between 5 and 18 years, and eight were 18 years and older. Patients stayed on average 7.0 days in the hospital (range 1 to 35) (Table 34). On average, iNTS patients were ill for 3.1 days before entering the hospital (0 to 10 days, standard deviation 3.8). No data was asked on the type of illness. 11 patients received care before entering the hospital, all from only one provider (Table 35). The most used providers were over-the-counter drugs and a health centre.

**Table 34: Hospital stay duration of iNTS patients**

| **Characteristic** | **Unit** | **Answer** | **Value** |
| --- | --- | --- | --- |
| Duration hospital stay | Days | Mean | 7.0 |
|  |  | Standard deviation | 6.8 |
|  |  | Range | 1 - 35 |

**Table 35: Providers of care to the iNTS patients before the patients entered the hospital**

| **Care provider** | **Number of patients** |
| --- | --- |
| Traditional healer | 1 |
| Over-the-counter drugs | 6 |
| Health facility/health post | 1 |
| Health centre | 3 |
| **Total** | **11** |

Admission diagnosis of 11 of the patients was related to an enteric bacterial pathogen, and 5 to diarrhoea and food intoxication each (Table 36). At discharge, eight patients were fully recovered and the rest was discharged with medical advice or referred to another hospital (Table 37). On the patient records of all except one patient it was recorded that they had comorbidities, either enteric bacterial pathogens or diarrhoea (Table 38). Although these were recorded as comorbidities, these seem to be coinfections.

**Table 36: Admission of the iNTS patients**

| **Admission diagnosis** | **Number of patients** |
| --- | --- |
| Food intoxication | 5 |
| Diarrhoea | 5 |
| Enteric bacterial pathogen | 11 |
| **Total** | **21** |

**Table 37: Health outcome on discharge of the iNTS patients.**

| **Health outcome on discharge** | **Number of patients** |
| --- | --- |
| Alive and well | 8 |
| Alive, but outcome unknown/missing | 13 |
| **Total** | **21** |

**Table 38: Type and number of comorbidities recorded on the iNTS patients’ records.**

| **Comorbidity** | **Number of patients** |
| --- | --- |
| No comorbidities | 1 |
| Diarrhoea | 5 |
| Enteric bacterial pathogen | 15 |
| **Total** | **21** |

Patients stayed on average in total 5.9 days (standard deviation 2.2, range 3 to 10 days) in the hospital (Table 39). They stayed on average 2.6 days in the intensive care unit, 1.8 days in the emergency room, 0.9 days in the pediatric ward, and 0.6 days in the outpatient clinic. Although an outpatient ward is only for patients that are not hospitalized, several patients stayed a full day or more in the outpatient ward, indicating they were hospitalized. When there was a shortage of beds on other wards/units for inpatients, the hospital used beds on the outpatient ward for such inpatients. However, on the patient record it was recorded that the patient stayed in the outpatient ward for that period. We have no data on the order in which patients stayed in each ward or unit.

**Table 39: Duration of stay in different wards of the iNTS patients (in days).**

| **Ward/unit ^1^** | **Mean (st.dev. ^2^)** | **Range** |
| --- | --- | --- |
| Outpatient clinic | 0.6 (1.3) | 0 - 5 |
| Pediatric ward | 0.9 (1.5) | 0 - 5 |
| Intensive care unit | 2.6 (3.0) | 0 - 7 |
| Emergency room | 1.8 (2.2) | 0 - 5 |
| Total stay | 5.9 (2.2) | 3 - 10 |

^1^ No stays at isolation unit or other locations. ^2^ Standard deviation.

All patients used a specialist consultation, ranging from 1 to 8 times (Table 40). All patients had a special diet, used specialist consultation, and almost all used intravenous fluids. Cardiopulmonary resuscitation and autopsy were not used. All patients used at least one diagnostic test (Table 41). Most diagnostic tests were only applied once for a patient.

**Table 40: Number of iNTS patients that used special services.**

|  | **Number of times used** | |
| --- | --- | --- |
| **Special service** | **Did not use** | **1** |
| Special diet | 0 | 21 |
| Specialist consultation | 0 | 21 |
| Intravenous fluids | 1 | 20 |
| Cardiopulmonary resuscitation | 21 | 0 |
| Autopsy | 21 | 0 |

**Table 41: Number of iNTS patients that used diagnostic tests.**

|  | **Number of times performed** | |
| --- | --- | --- |
| **Diagnostic test** | **Did not use** | **1** |
| Blood count |  |  |
| - full count | 2 | 19 |
| - Hb | 2 | 19 |
| - HCT/PCV | 2 | 19 |
| Blood culture | 21 |  |
| Bone marrow culture | 21 |  |
| Widal agglutination | 21 |  |
| Tubex TF | 21 |  |
| Typhidot TF Elisa | 21 |  |
| RTI | 21 |  |
| Antigen | 21 |  |
| PCR | 21 |  |
| HIV test |  |  |
| - rapid test | 21 |  |
| - Elisa | 21 |  |
| Other microbiological tests |  |  |
| - gram stain/Microscopy |  | 21 |
| - culture |  | 21 |
| - sensitivity |  | 21 |
| - antigen |  | 21 |
| Radiology test: |  |  |
| - CT scan | 21 |  |
| - Ultrasound | 21 |  |
| - Other X-ray | 21 |  |
| Blood chemistry test |  |  |
| - Electrolytes | 5 | 16 |
| - Glucose | 5 | 16 |

Microbiological test results recorded on the patient records showed that all patients diagnosed with iNTS had *Salmonella arizonae* (Table 42). All patients received antibiotics. Four different types of antibiotics were administered with different routes, doses, frequency, and number of days (Table 43). No complications were observed. Table 44 shows that all 21 iNTS patients used ambulance service.

**Table 42: Microbiological test results of iNTS patients.**

| **Test results** | **Number of patients** |
| --- | --- |
| *Salmonella arizonae* | 21 |
| **Total** | **21** |

**Table 43: Number of iNTS patients that used drugs.**

|  |  |  |  |  | **Number of days** | |
| --- | --- | --- | --- | --- | --- | --- |
| **Drug** | **Route ^1^** | **Dose** | **Unit** | **Frequency** | **10** | **14** |
| Azithromycin | I | 4 | g | Daily |  | 16 |
| Azithromycin | I | 200 | mg | Daily | 4 |  |
| Ceftriaxone | I | 1 | g | BID | 10 | 3 |
| Ciprofloxacin | I | 200 | mg | Daily | 9 |  |
| Trimethoprim | O | 16 | mg | Daily | 1 | 7 |

^1^ I=intravenous, O=oral.

##

**Table 44: Number of iNTS patients that used the ambulance service.**

|  | **Did not use** | **1 time** |
| --- | --- | --- |
| Ambulance service | 0 | 21 |
