# Supplementary material for: Patient-related healthcare costs for diarrhoea, Guillain Barré syndrome and invasive non-typhoidal salmonellosis in Gondar, Ethiopia, 2020
Source: BMC Public Health. 2022 Nov 16;22:2091. doi: 10.1186/s12889-022-14539-1 (PMC9670532; doi:10.1186/s12889-022-14539-1)
Supplement: Supplementary file 4 — Additional file 4. Supplementary material S4. Median direct medical costs per diarrhoeapatient by etiology and healthcare facility. [file 12889_2022_14539_MOESM4_ESM.docx]

**Supplementary material S4 to Direct and indirect (non)-medical patient-related healthcare costs for diarrhoea, GBS, and iNTS in three healthcare facilities in Gondar, Ethiopia, 2020**

**Median direct medical costs per diarrhoea patient by etiology and healthcare facility**

**Median direct medical costs in 2020 USD per diarrhoea patient by etiology^1^ in three healthcare facilities in Gondar, Ethiopia, in 2020.**

|  |  | **Etiology^2^** | | |  |
| --- | --- | --- | --- | --- | --- |
| **Healthcare facility** | **Cost category** | ***Campylobacter* spp.** | **enterotoxigenic *Escherichia coli*** | **non-typhoidal *Salmonella enterica*** | **p-value^3^** |
| All facilities | Total costs | 9.85 | 9.16 | 8.59 | 0.390 |
|  | Stay costs | 1.15 | 0.57 | 0.57 | 0.306 |
|  | Special services costs | 2.29 | 2.29 | 2.00 | 0.162 |
|  | Diagnostic test costs | 2.43 | 2.29 | 2.29 | 0.103 |
|  | Drug costs | 3.15 | 3.15 | 3.03 | 0.301 |
| Health centre | Total costs | 6.33 | 5.73 | 6.99 | 0.498 |
|  | Stay costs | 0.43 | 0.43 | 0.43 | 0.987 |
|  | Special services costs | 1.57 | 1.86 | 1.86 | 0.517 |
|  | Diagnostic test costs | 0.72 | 0.72 | 0.72 | 0.222 |
|  | Drug costs | 3.09 | 2.58 | 4.01 | 0.304 |
| Private clinic | Total costs | 10.42 | 10.59 | 9.56 | 0.177 |
|  | Stay costs | 1.43 | 1.43 | 1.43 | 0.181 |
|  | Special services costs | 2.29 | 4.01 | 2.00 | 0.567 |
|  | Diagnostic test costs | 2.43 | 2.43 | 2.43 | 0.952 |
|  | Drug costs | 2.55 | 2.19 | 2.19 | 0.873 |
| Specialised hospital | Total costs | 10.99 | 9.16 | 8.59 | 0.075 |
|  | Stay costs^3^ | 0.57 | 0.57 | 0.57 | 0.106 |
|  | Special services costs | 2.58 | 2.29 | 2.00 | 0.243 |
|  | Diagnostic test costs | 3.15 a | 2.86 a | 2.29 b | 0.007 |
|  | Drug costs | 3.89 | 3.15 | 3.15 | 0.099 |

^1^ Analysis has been performed for the pathogens of interest for our study. ^2^ Different letters between columns indicate a difference in medians of transformed costs at p < 0.05 between pathogens as tested with pairwise comparison with adjusted p-value. ^3^ Chi squared test to test for differences in median of transformed costs.
